# Potential Role of Semaphorin 3A and Its Receptors in Regulating Aberrant Sympathetic Innervation in Peritoneal and Deep Infiltrating Endometriosis

**DOI:** 10.1371/journal.pone.0146027

**Published:** 2015-12-31

**Authors:** Yanchun Liang, Wei Wang, Jiaming Huang, Hao Tan, Tianyu Liu, Chunliang Shang, Duo Liu, Luyan Guo, Shuzhong Yao

**Affiliations:** Department of Obstetrics and Gynecology, First Affiliated Hospital of Sun Yat-sen University, No. 58, the 2nd Zhongshan Road, Yuexiu District, Guangzhou city, Guangdong Province, 510080, China; Rutgers University, UNITED STATES

## Abstract

Previous studies have demonstrated the involvement of nerve repellent factors in regulation of the imbalanced innervation of endometriosis. This prospective study aims to explore the role of Sema 3A in regulating aberrant sympathetic innervation in peritoneal and deep infiltrating endometriosis. Ectopic endometriotic lesion were collected from patients with peritoneal endometriosis (n = 24) and deep infiltrating endometriosis of uterosacral ligament (n = 20) undergoing surgery for endometriosis. Eutopic endometrial samples were collected from patients with endometriosis (n = 22) or without endometriosis (n = 26). Healthy peritoneum (n = 13) from the lateral pelvic wall and healthy uterosacral ligament (n = 13) were obtained from patients who had no surgical and histological proof of endometriosis during hysterectomy for uterine fibroids. Firstly, we studied the immunostaining of Sema 3A, Plexin A1 and NRP-1 in all the tissues described above. Then we studied the nerve fiber density (NFD) of endometriosis-associated (sympathetic) nerve and para-endometriotic (sympathetic) nerve by double immunofluorescence staining. Finally we analyzed the relationship between expression of Sema 3A in stromal cells of endometriotic lesion and the aberrant innervation of endometriosis. Semi-quantitative immunostaining demonstrated that (1) Higher immunostaining of Sema 3A were found in the eutopic endometrial glandular epithelial cells from patients with endometriosis (p = 0.041) than those without endometriosis; (2) Sema 3A immunostaining was higher in glandular epithelial cells of peritoneal endometriosis (P<0.001) and deep infiltrating endometriotic lesions of uterosacral ligament (P = 0.028)compared with glandular epithelial cells of the endometrium from women with endometriosis, while its expression in ectopic stormal cells in both groups were significantly lower than that from eutopic endometrium of women without endometirosis (P<0.001, P<0.001, respectively). NFDs of Anti-TH (+) endometriosis-associated sympathetic nerve of peritoneal endometriosis (p<0.001) and deep endometriosis of uterosacral ligament (p<0.001) were significantly lower than NFDs of para-endometriotic sympathetic nerve. Our results suggest that Sema 3A may contribute to the regulation of aberrant sympathetic innervation in peritoneal and deep infiltrating endometriosis.

## Introduction

Endometriosis is a chronic, inflammatory, estrogen-dependent benign gynecological disease characterized by the presence of endometrial glands and stromal cells outside the uterine cavity. It is often associated with infertility and pelvic pain, affecting 10–15% of women of childbearing age worldwide [1]. The relationship between endometriosis and pain is still poorly understood. And the mechanisms underlying endometriosis-associated-pain remain to be further investigated.

Recent studies have shown endometriotic lesions develop newly formed sensory and autonomic nerve fibers in rat models and in women with endometriosis [2–4], often in close contact with endometriotic implants and accompany with the immature blood vessels that vascularize the ectopic growth [5]. Tokushige et al. demonstrated that protein gene product (PGP) 9.5-immunoactive nerve fibers were present in peritoneal or deep infiltrating endometriotic lesions [1]. The density of endometriosis-associated nerve fibers were correlative with the severity of pain in women with endometriosis [6], suggesting a direct association between pain and nerve fiber density.

An imbalance in sympathetic/sensory innervation in the inflamed area in women with peritoneal and intestinal endometriosis was demonstrated recently. They found significant loss of sympathetic innervation in the area near the peritoneal and intestinal endometriotic lesions, where the sensory nerve density remained unchanged or increased [7–8]. This aberrant sympathetic and sensory innervation is proposed to be an adaptive program in order to maintain the balance of pro-inflammatory and anti-inflammatory effects. This is in consistent with the observations in other autoimmune and chronic inflammatory disease, such as rheumatoid arthritis and Crohn disease [9–10]. However, the underlying pathophysiology of this interesting phenomenon is not well understood. Studies have shown that several nerve repellent factors (Semaphorin 3A, Semaphorin 3C, Semaphorin 3F, neuropilin 2, etc.) may play an important role in regulating the imbalanced innervation of different inflammatory diseases [11–13]. Accordingly, we speculate that semaphorin 3A (Sema 3A), a member of these nerve repellent factors, may also be involved in modulating the aberrant innervation within endometriotic lesions.

Semaphorins are a group of evolutionarily highly conserved surface or locally secreted nerve repellent factors that not only steer and fasciculate axons in the developing nervous system [14] but also regulate both developmental [14] and tumor angiogenesis [15–17]. Sema 3A, a member of secreted class 3 semaphorins, is well reported as a potent chemorepellent that restricts axonal elongation and causes growth cone collapse [18] through binding to its specific receptor, neuropilin-1 (NRP-1), which is located on the surface of the target neurons. It is suggested that NRP-1 must bind with Plexin A1 [19], another receptor to Sema 3A, in order to form a multimeric holoreceptor signaling complex that triggers the down-stream signaling cascade [20–22]. Tolofari et al. demonstrated that with the reduced expression of Sema 3A in degenerated intervertebral disc, there was a disinhibition of neural ingrowth, thus resulting in increased density of PGP9.5 positively stained nerve fibers in the degenerative disc [23]. In addition, the increased uterine Sema 3A expression during pregnancy is associated with reduced sympathetic innervation [24]. Sema 3A has also been strongly implicated as an inhibitory factor determining density of sympathetic innervation of blood vessels [25]. It was reported that pro-inflammatory factors initiating up-regulation of Sema 3A was associated with decreased density of sympathetic and sensory nerve fibers in benign colorectal adenomatous polyps [13].

As is known to all, endometriosis is a chronic pelvic inflammatory disease. Many studies have shown that different expression of various cytokines or chemokines as well as infiltration of inflammatory cells in peritoneal microenvironment. And endometriotic lesions contribute to the inflammatory condition of endometriosis. We hypothesize that Sema 3A, a nerve repellent factor, may play a potential role in regulating the aberrant sympathetic innervation in peritoneal and deep infiltrating endometriosis. The present study tried to examine the expression of the chemorepulsive Sema 3A and its receptors (NRP-1 and Plexin A1) in peritoneal and deep infiltrating endometriosis first. And then we further investigated the distribution of PGP9.5-immunoactive or Tyrosine Hydroxylase-immunoactive sympathetic nerve fibers in/near the endometriotic lesions and analyze the association between expression of Sema 3A and the density of these nerve fibers.

## Material and Methods

### Patients

The study was conducted in the Department of Obstetrics and Gynecology of the First Affiliated Hospital of Sun Yat-sen Universigy from December 2012 to January 2014. Tissue samples consisted of 24 peritoneal endometriotic tissues and 20 deep infiltrating endometriotic tissues (all of which are from uterosacral ligaments) from 44 premenopausal women requiring surgical treatment. Endometrial tissue samples were collected from the same patient in 22 cases (women with endometriosis, secretory phase n = 8 and proliferative phase n = 14). The mean ages of patients according to the type of tissue collected were: endometriotic lesions 32 years (range 23–43 years), eutopic endometrium from women with endometriosis 34 years (range 26–40 years). And all of the patients had a regular menstrual cycle (secretory phase n = 18 and proliferative phase n = 26), without malignant or inflammatory diseases. The presence of endometrial glands and surrounding stromal cells was regarded as histological proof of endometriosis.

In addition, normal endometrial samples were obtained from 26 women (mean age: 37 years; range: 33–47 years; secretory phase n = 10 and proliferative phase n = 16) undergoing hysterectomy or endometrial biopsy for benign diseases, either uterine fibroids or infertility. Healthy peritoneum (n = 13) from the lateral pelvic wall and healthy uterosacral ligament (n = 13) were obtained from patients (mean age: 39 years; range: 34–49 years) with a regular menstrual cycle (secretory phase n = 12 and proliferative phase n = 14). Patients who had no surgical and histological proof of endometriosis during hysterectomy (laparoscopy or laparotomy) for uterine fibroids were included in these groups. The lower basis of all peritoneal biopsies was the sub-peritoneal fat. The uterosacral ligaments were sampled 1 cm from the uterus.

None of the patients had received hormonal treatment for at least 3 months prior to surgery. All of the patients had no malignant or inflammatory disease. All the tissue samples were obtained with written, full and informed consent from all the patients. The research protocol was approved by the Research Ethics Committee of the First Affiliated Hospital of Sun Yat-sen University.

### Antibodies and immunohistochemistry

Tissues were fixed immediately in formalin (10%) and then processed as paraffin blocks. Four micrometer-thick sections of formalin-fixed tissues were deparaffinated in xylene and rehydrated through a graded series of ethanol solutions. The initial section was stained with hematoxylin-eosin for tissue diagnosis. Immunohistochemical staining was performed on paraffin sections with rabbit polyclonal antibodiy directed against Sema 3A (dilution 1:50, Sigma), rabbit polyclonal antibodiy against NRP-1 (dilution 1:20, Sigma), rabbit polyclonal antibodiy against Plexin A1 (dilution 1:50, Sigma). The antigen retrieval was performed in a pressure cooker in a sodium citrate buffer (10 mM, pH 5.5) for 2 minutes (start the time as soon as the cooker has reached full pressure) using SP Rabbit HRP kit (CWBIO Tech, Beijing, China). The sections were incubated with endogenous peroxidase for 10 minutes, washed three times with phosphate buffered saline (PBS, pH7.4) and then blocked with 5% normal goat serum for 30 minutes. After Washing with PBS, the sections were incubated with primary antibodies against Sema 3A, NRP-1 and Plexin A1 overnight at 4°C. After washing with PBS, the sections were incubated with secondary antibodies for 1 hour at room temperature and then washed with PBS three times. Detection of bound antibody was with 1- to 5-minute incubation at room temperature with 3, 3’-diaminobenzidine (DAB) substrate. Slides that were used as specificity controls underwent the same procedure without primary antibodies. These procedures resulted in negative staining. Positive controls are presented in S1, S2 and S3 Figs. The histological slides were scanned using an Olympus BX51 microscope (Tokyo, Japan) and microscopic images containing mostly stained epithelial cells were captured and digitized using a digital camera connected to a computer. Five mostly stained microscopic fields at a magnification of ×200 were included in the measurement.

### Analysis of immunohistochemical results

For qualitative analysis, samples were considered negative when no labelled cells were observed on the tissue section and positive in all other cases. The immunohistochemical result was scored in a semiquantitative fashion incorporating both the intensity of specific staining and the percentage of stained epithelial cells. The intensity of the staining was evaluated as follows: 0 = no, 1 = weak, 2 = moderate and 3 = intense immunostaining. The percentage of stained epithelial cells was calculated by two independent experienced pathologists. Differences in opinion between the observers were resolved at a discussion microscope.

For each observed slide, we used a calculated value known as HSCORE for further analysis.

$$\text{HSCORE}=\sum {\text{P}}_{\text{i}}\left(\text{i}+1\right),$$

where i is the intensity of the staining varying from 0 to 3 and P_i_ is the percentage of stained cells [26]. Each slide was examined at least twice.

### Double immunofluorescence staining and quantification of nerve fiber density

Immunofluorescence histochemical double staining technique was used to identify the target nerve fibers. Antibodies against protein gene product 9.5 (Anti-PGP 9.5, monoclonal mouse, dilution 1:500, Abcam, UK), tyrosine hydroxylase (Anti-TH, monoclonal mouse, dilution 1:500, Abcam, UK) were used to identify total nerve fibers and sympathetic nerve fibers, respectively. Antibody against Sema 3A (polyclonal rabbit, dilution 1:50, Abcam, UK) was used to identify the endometrial epithelial cells. The procedures before the incubation of primary antibody were the same as the immunohistochemical staining described above. Incubate the sections in the mixture of two primary antibodies (rabbit against Sema 3A and mouse against PGP 9.5 or TH) in 1% BSA in PBS in a humidified chamber overnight at 4°C. Wash the sections for three times with PBS and then incubate the sections with the mixture of two secondary antibodies which are raised in different species (DyLight 488 AffiniPure Goat Anti-Rabbit IgG and DyLight 549 AffiniPure Goat Anti- Mouse IgG, EarthOx, LLC, San Francisco, CA, USA) in 1% BSA for 1 hour at room temperature in dark. Decant the mixture of the secondary antibody solution and wash three times with PBS for 5 minutes each in dark. DAPI staining solution (Sigma) is used to identify the neuclei (incubate for 10 minutes). Rinse the slides with PBS (3×5 minutes). Drain excess buffer from the coverslip and mount with a mounting medium containing an antifade reagent (Product number: ab104136, Abcam, UK). The double immunofluorescence stained slides were scanned using an Olympus BX51 microscope (Tokyo, Japan) and microscopic images were captured and digitized using a digital camera connected to a computer. Five clearly stained microscopic fields at a magnification of ×200 were included in the measurement.

Under microscope, the endometrial epithelial cells were markedly labelled by green fluorescence, while the nerve fibers were labelled by orange-yellow fluorescence. Any nerve found within 1.5 mm of an endometriotic lesion was defined as endometriosis-associated nerve (EAN) [27], while the nerve found in the area (with a normal histological tissue) at least 4-mm distance from the endometriotic lesion was defined as para-endometriotic nerve (PEN) [8]. The number of nerve fibers per square millimeter (nerve fiber density, NFD) was determined by averaging the number of counted nerve fibers, using a grid of 1 mm^2^, in 5 non-overlapping indiscriminately selected high-power fields (magnification ×200) [28]. All of the slides were examined and the NFD were calculated blindly by two independent investigators. Finally, the average NFDs of the results from two investigators were employed for further analysis.

### Severity of dysmenorrhea (evaluated by VAS)

Using a standardized questionnaire with a visual analogue scale (VAS), the degree of dysmenorrhea was documented before surgery. In VAS evaluation, the degree of dysmenorrhea was quantified on a scale of 0–100 mm. “No pain” was indicated at the left side of the scale and “the maximum pain you could imagine” at the right side of the scale. The value of VAS was collected by the investigator responsible for preoperative data collection. The severity of dysmenorrhea was defined as the following: 1–3 points (mild pain), 4–6 points (moderated pain), and 7–10 points (severe pain).

### Statistical analysis

The Kruskal-Wallis and Mann-Whitney tests were used for comparisons of semi-quantitative immunostaining HSCORE and NFDs. Statistical significance was defined *P*<0.05. Statistical analysis was performed using GraphPad Prism 5 for Windows (GraphPad Software, 2003, USA). Means and standard deviations are shown.

## Results

### Qualitative and semi-quantitative immunostaining of Sema 3A, NRP-1 and Plexin A1 in the endometrium

**Fig 1:**
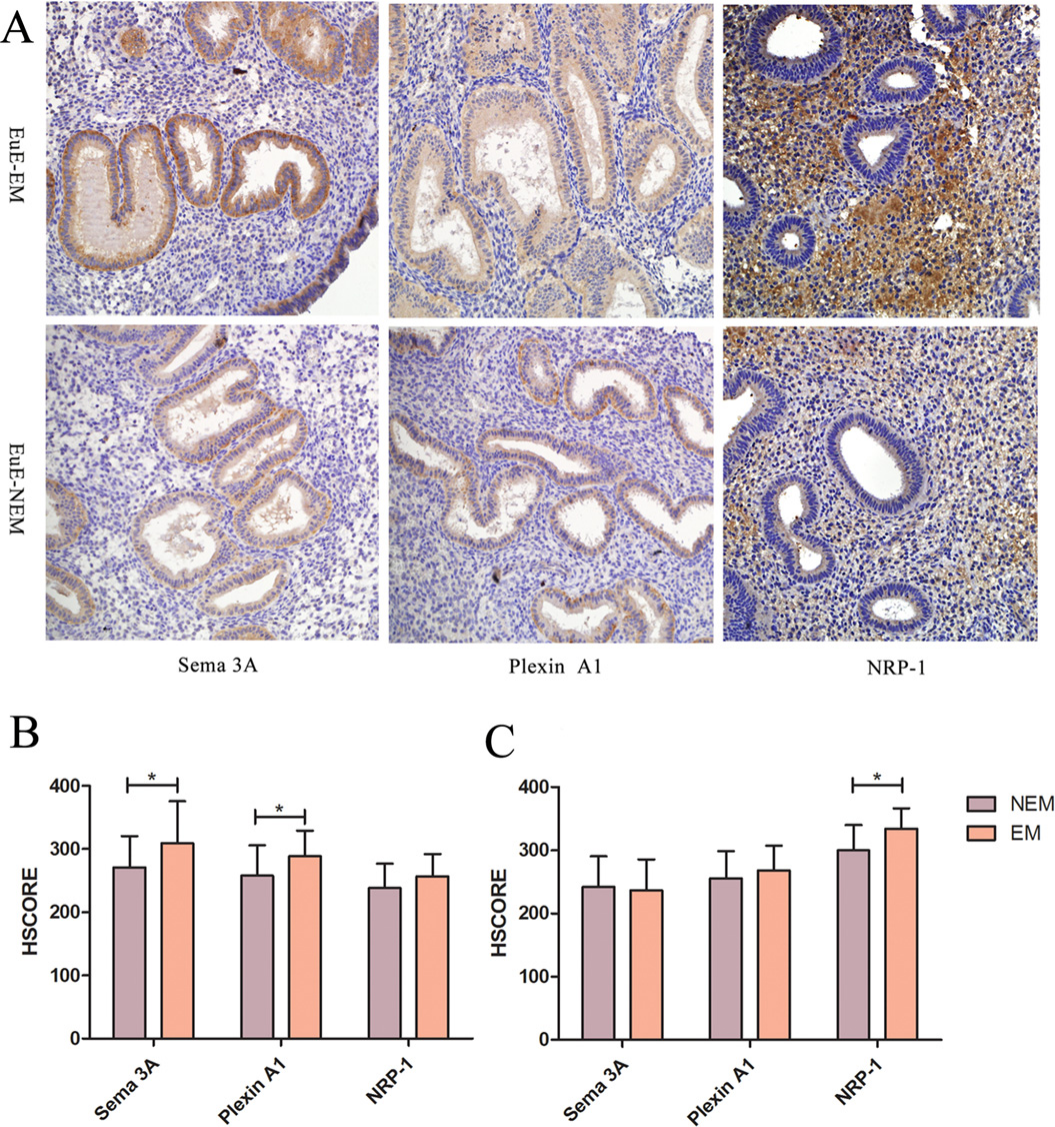
The expression of Sema 3A, Plexin A1 and NRP-1 in eutopic endometrium from patients with endometriosis and patients without endometriosis. A: Immunohistochemical staining of three proteins in eutopic endometrium. EuE-EM: Eutopic Endometrium from patients with Endometriosis; EuE-NEM: Eutopic Endometrium from patients without Endometriosis (immunohistochemical stain, 200 × magnification). B and C: Semi-quantitative expression of Sema 3A, Plexin A1 and NRP-1 of glandular epithelial cells (B) as well as stromal cells (C) from eutopic endometrium from patients with endometriosis (EM) and without endometriosis (NEM). *P<0.05 (Mann–Whitney).

Sema 3A, NRP-1 and Plexin A1 were expressed in both the endometrial glandular epithelial cells and stromal cells (Fig 1A, S4, S5, S6 and S7 Figs). For qualitative immunostaining, Sema 3A, NRP-1 and Plexin A1 were stained positively in all of the detected sections. Higher immunostaining of Sema 3A and Plexin A1 were found in the eutopic endometrial glandular epithelial cells from patients with endometriosis (p = 0.041 and p = 0.025, respectively) than those without endometriosis. But the expression of Sema 3A as well as Plexin A1 between the stromal cells from patients with endometriosis and those without endometriosis were not significantly different (p = 0.067 and p = 0.319, respectively). There was no significant difference between the HSCORE of NRP-1 of eutopic endometrial glandular epithelial cells from patients with endometriosis and that from patients without endometriosis (p = 0.120), but the HSCORE of NRP-1 of stromal cells from patients with endometriosis was obviously higher than that from patients without endometriosis (p = 0.002). The semi-quantitative immunostaining of Sema 3A, NRP-1 and Plexin A1 in the endometrial samples using HSCORE analysis is shown in S1 Table (Fig 1B and 1C).

### Qualitative and semi-quantitative immunostaining of Sema 3A, NRP-1 and Plexin A1 in peritoneal endometriosis and deep infiltrating endometriosis of uterosacral ligament

**Fig 2:**
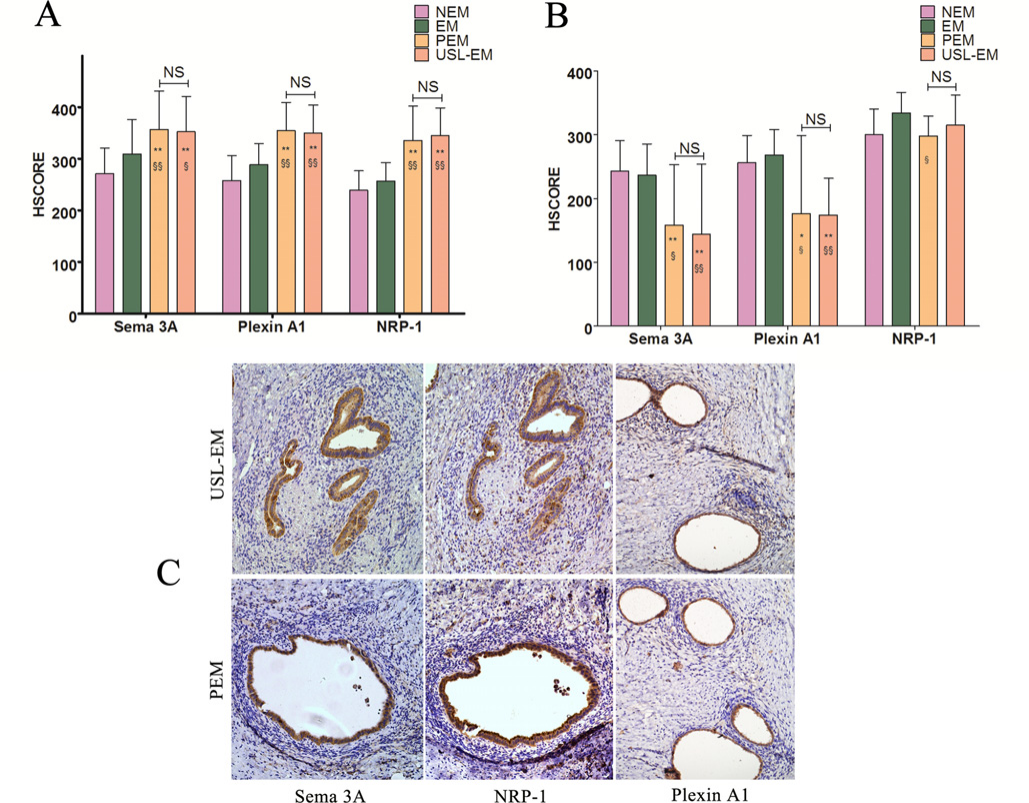
Expression of Sema 3A, Plexin A1 and NRP-1 in peritoneal endometriotic lesions and deep infiltrating endometriotic lesions of uterosacral ligament. A: Comparison of semi-quantitative expression of Sema 3A, Plexin A1 and NRP-1 of glandular epithelial cells between peritoneal endometriosis, deep infiltrating endometriosis of uterosacral ligament and eutopic endometrium from patients with endometriosis (EM) as well as without endometriosis (NEM). B: Comparison of semi-quantitative expression of Sema 3A, Plexin A1 and NRP-1 of stromal cells between peritoneal endometriosis, deep infiltrating endometriosis of uterosacral ligament and eutopic endometrium from patients with endometriosis (EM) as well as without endometriosis (NEM). C: Immunohistochemical staining of Sema 3A, Plexin A1 and NRP-1 in PEM and USL-EM. PEM: peritoneal endometriosis; USL-EM: deep infiltrating endometriosis of uterosacral ligament. (immunohistochemical stain, 200 × magnification) Mann-Whitney test: *P<0.05 (versus NEM); ** *P*<0.001 (versus EM); §*P*<0.05 (versus NEM); §§*P*<0.001(versus EM); NS, not significant.

Sema 3A, NRP-1 and Plexin A1 immunostaining were found 100% of peritoneal and deep infiltrating endometriosis samples (Fig 2C). Sema 3A immunostaining was higher in glandular epithelial cells of peritoneal endometriosis (HSCORE = 356.77±74.3) compared with glandular epithelial cells of the endometrium from women with endometriosis (HSCORE = 309.09±66.61, *P*<0.001) or glandular epithelial cells of the endometrium from women without endometriosis (HSCORE = 271.15±49.34, *P*<0.001, S12 Fig). The expression of both NRP-1 and Plexin A1 in glandular epithelial cells of peritoneal endometriosis were also higher than that from eutopic endometrium of women with endometriosis as well as women without endometriosis (all the p values were less than 0.001) (Fig 2A).

On the other hand, Sema 3A immunostaining in stromal cells of peritoneal endometriosis (S9 Fig) (HSCORE = 158.33±94.60) was significantly lower than that from eutopic endometrium of women with endometriosis (HSCORE = 236.36±49.24, *P* = 0.002) as well as women without endometriosis (HSCORE = 242.31±48.36, *P*<0.001) (Fig 2B). This lower expression was also observed for Plexin A1 (S11 Fig) (p = 0.005, p = 0.005, respectively). The expression of NRP-1 in stromal cells of peritoneal endometriosis was lower than the stromal cells of endometrium of women with endometriosis (p = 0.001), while there was no difference between stromal cells of peritoneal endometriosis and eutopic endometrium of women without endometriosis (p = 0.082).

Relative to glandular epithelial cells of deep infiltrating endometriotic lesions of uterosacral ligament, glandular epithelial cells of eutopic endometrium from women with or without endometriosis showed lower expression of Sema 3A (p = 0.028, p<0.001, respectively), NRP-1 (p<0.001, p<0.001, respectively) and Plexin A1 (p<0.001, p<0.001, respectively) (Fig 2A). Similarly, Sema 3A immunostaining in stromal cells of deep infiltrating endometriosis (S8 Fig) (HSCORE = 143.75±109.99; p<0.001, p<0.001, respectively) was lower than that from eutopic endometrium of women with endometriosis as well as women without endometriosis. This lower expression was also observed for Plexin A1 (S10 Fig) (p<0.001, p<0.001, respectively). But no differences were observed for NRP-1 (Fig 2B).

### Total nerve fiber density and sympathetic nerve fiber density in peritoneal endometriotic specimens and healthy peritoneum

PGP 9.5 is a highly specific pan-neuronal marker for both myelinated and unmyelinated nerve fibers, including Aα, Aβ, Aγ, Aδ, B and C fibers [29], while Tyrosine Hydroxylase (TH) is a specific marker for sympathetic nerve fibers. Anti-PGP 9.5 and Anti-TH positively stained nerve fibers were observed in peritoneal endometriotic lesions, para-endometriotic tissue and healthy peritoneum (S13 Fig).

**Fig 3:**
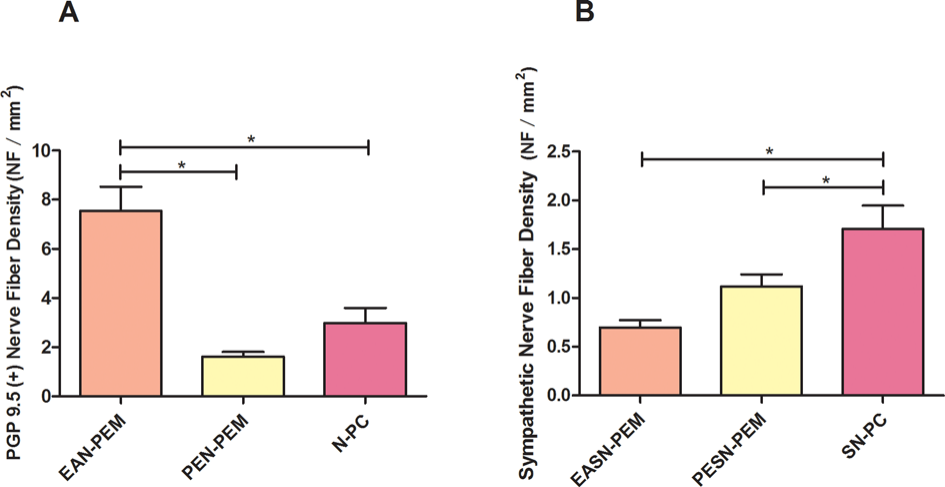
Comparison of total nerve fiber density and sympathetic nerve fiber density in peritoneal endometriotic specimens and healthy peritoneum. (A) Total nerve fibers were positively stained by Anti-PGP 9.5 antibody. Comparison of the total nerve fiber density (NFD, NF/mm^2^) of endometriosis-associated nerve of peritoneal endometriosis (EAN-PEM), NFD of para-endometriotic nerve of peritoneal endometriosis (PEN-PEM) and NFD of nerve of peritoneum of control (N-PC). (B) Sympathetic nerve fibers were positively stained by Anti-TH antibody. Comparison of endometriosis-associated sympathetic NFD of peritoneal endometriosis (ESAN-PEM), para-endometriotic sympathetic NFD of peritoneal endometriosis (PESN-PEM) and sympathetic NFD of peritoneum of control (SN-PC). *P<0.05.

The nerve fiber density (NFD) of Anti-PGP 9.5 (+) endometriosis-associated nerve of peritoneal endometriosis (EAN-PEM) was significantly higher than NFD of para-endometriotic nerve of peritoneal endometriosis (PEN-PEM) and NFD of nerve of peritoneum of control (N-PC) (p<0.001, p = 0.002, respectively) (Fig 3A, S3 Table). NFD of Anti-TH (+) endometriosis-associated sympathetic nerve of peritoneal endometriosis (EASN-PEM) was significantly lower than NFD of para-endometriotic sympathetic nerve of peritoneal endometriosis (PESN-PEM) (p<0.001). And NFD of PESN-PEM was also lower than that of sympathetic nerve of peritoneum of control (SN-PC) (p = 0.028) (Fig 3B, S4 Table).

### Total nerve fiber density and sympathetic nerve fiber density in deep infiltrating endometriotic specimens of uterosacral ligament and healthy uterosacral ligament

Similarly, Anti-PGP 9.5 and Anti-TH positively stained nerve fibers were observed in uterosacral endometriotic lesions, para-endometriotic tissue and healthy uterosacral ligament (S13 Fig).

**Fig 4:**
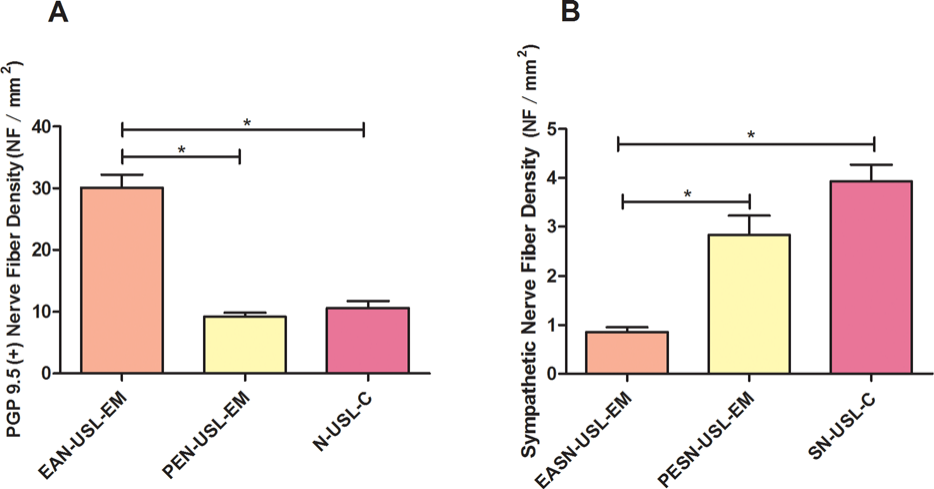
Comparison of total nerve fiber density and sympathetic nerve fiber density in deep infiltrating endometriotic specimens of uterosacral ligament and healthy uterosacral ligament. (A) Total nerve fibers were positively stained by Anti-PGP 9.5 antibody. Comparison of the total nerve fiber density (NFD, NF/mm^2^) of endometriosis-associated nerve of deep infiltrating endometriosis of uterosacral ligament (EAN-USL-EM), NFD of para-endometriotic nerve of deep infiltrating endometriosis of uterosacral ligament (PEN-USL-EM) and NFD of nerve of uterosacral ligament of control (N-USL-C). (B) Sympathetic nerve fibers were positively stained by Anti-TH antibody. Comparison of endometriosis-associated sympathetic NFD of USL-EM (ESAN-USL-EM), para-endometriotic sympathetic NFD of USL-EM (PESN-USL-EM) and sympathetic NFD of uterosacral ligament of control (SN-USL-C). *P<0.05.

**Fig 5:**
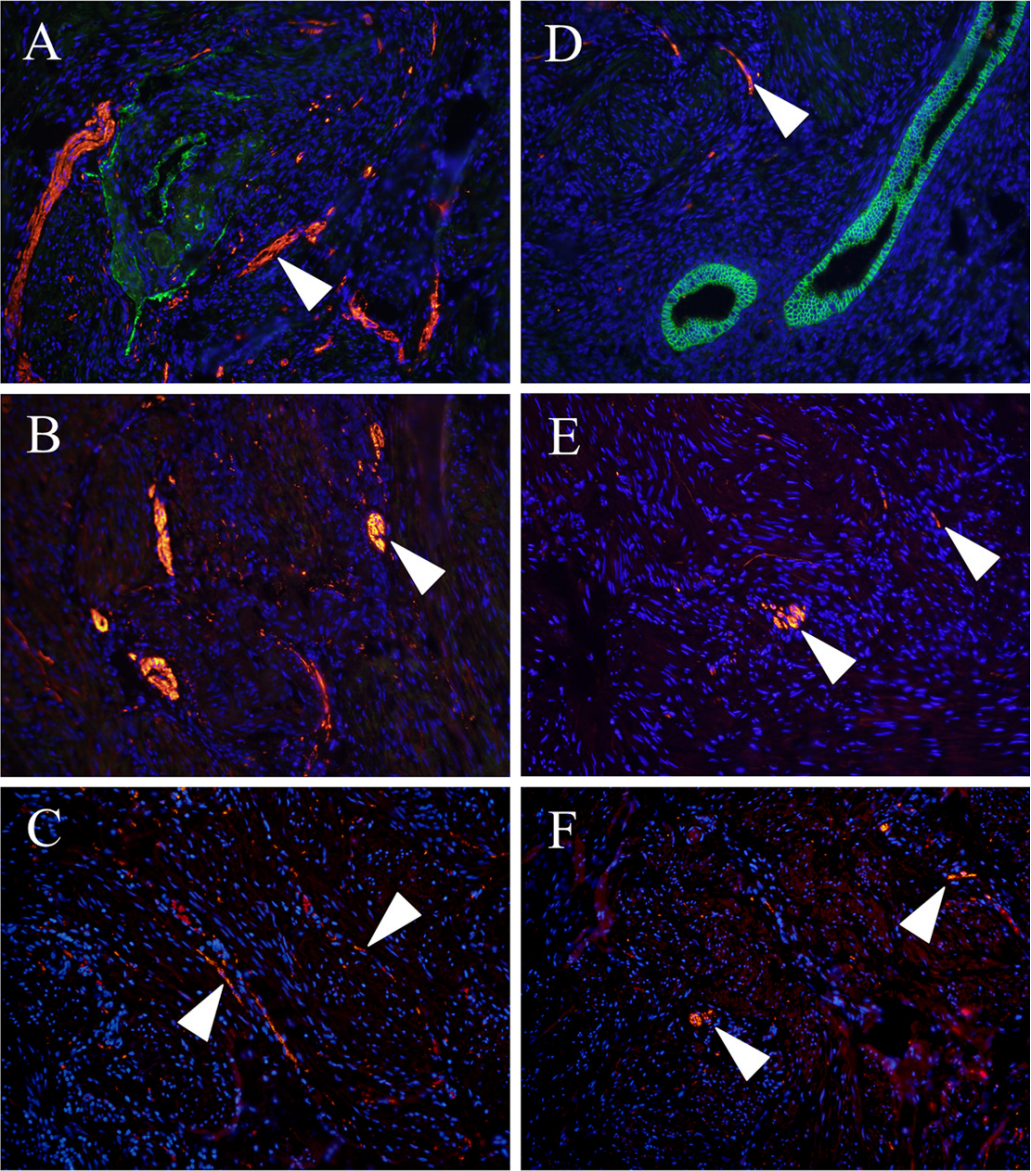
Immunofluorescence histochemical staining of the total nerve fiber and sympathetic nerve fiber in deep infiltrating endometriotic specimens of uterosacral ligament and healthy uterosacral ligament. Total nerve fibers were orange-yellow stained by Anti-PGP 9.5 antibody (A: EAN-USL-EM; B: PEN-USL-EM; C: N-USL-C). Sympathetic nerve fibers were also orange-yellow stained by Anti-TH antibody (D: EASN-USL-EM; E: PESN-USL-EM; F: SN-USL-C). Ectopic endometrial glandular epithelial cells was stained in green by anti-Sema 3A antibody staining (A was the merge image of double staining of both Sema 3A and PGP 9.5, D was the merge image of double staining of both Sema 3A and TH), nuclei were stained in blue by DAPI staining. White triangle: orange-yellow stained nerve fibers. (Original magnification 200×)

The NFD of Anti-PGP 9.5 (+) endometriosis-associated nerve of uterosacral ligament endometriosis (EAN-USL-EM) was significantly higher than NFD of para-endometriotic nerve of uterosacral ligament endometriosis (PEN-USL-EM) as well as NFD of nerve of uterosacral ligament of control (N-USL-C) (p<0.001, p<0.001, respectively) (Fig 4A, S5 Table). NFD of Anti-TH (+) endometriosis-associated sympathetic nerve of uterosacral ligament endometriosis (EASN-USL-EM) was also lower than NFD of para-endometriotic sympathetic nerve of uterosacral ligament endometriosis (PESN-USL-EM) (p<0.001). And NFD of PESN-USL-EM was also lower than that of sympathetic nerve of uterosacral ligament of control (SN-USL-C) (p = 0.012) (Fig 4B, S6 Table) (Fig 5).

### Relationship between Sema 3A expression in stromal cells, total endometriosis-associated nerve fiber density and severity of dysmenorrhea

**Fig 6:**
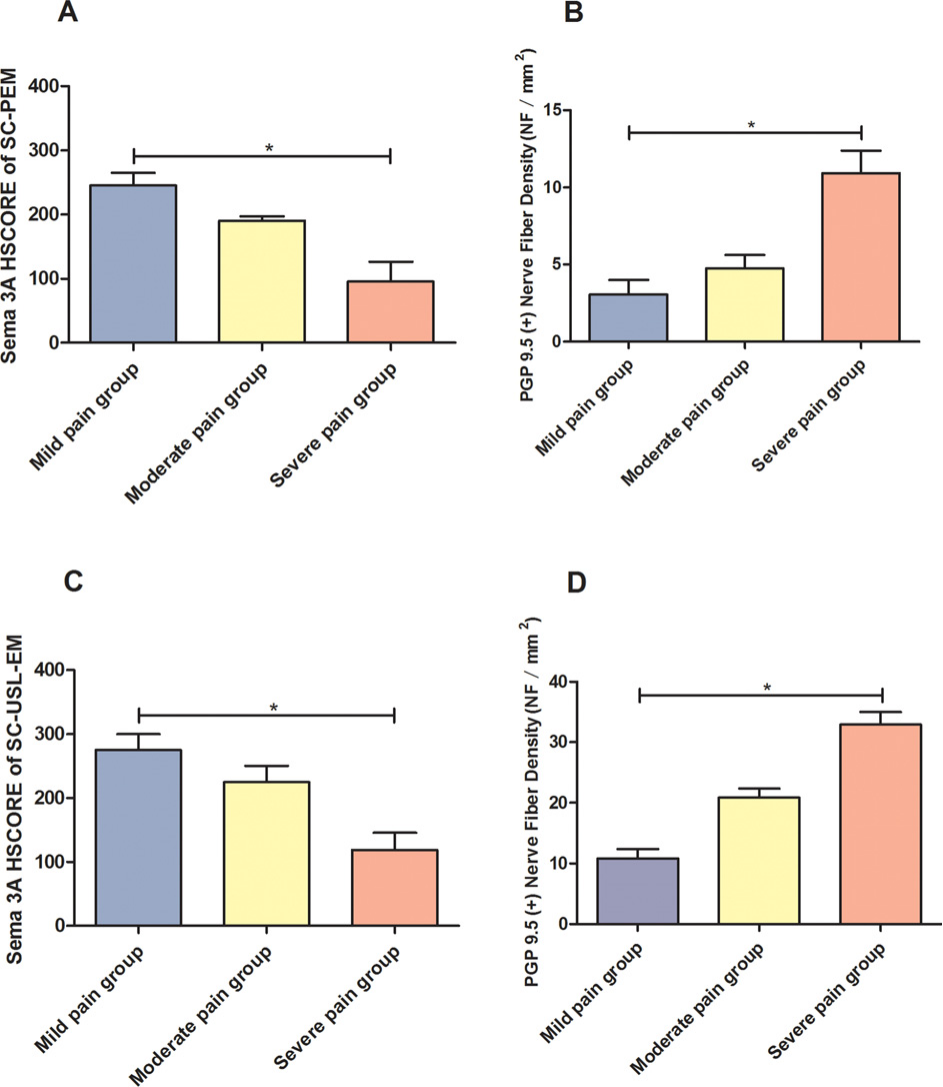
Relationship between immunostaining of Sema 3A in stromal cells of endometriosis, total endometriosis-associated nerve fiber density and severity of dysmenorrhea. A and B: Patients with higher pain VAS score presents with lower Sema 3A HSCORE of stromal cells of peritoneal endometriosis (SC-PEM), but with higher PGP 9.5 (+) total nerve density. C and D: Patients with higher pain VAS score presents with lower Sema 3A HSCORE of stromal cells of deep infiltrating endometriosis of uterosacral ligament (SC-USL-EM), but with higher PGP 9.5 (+) total nerve fiber density. *P<0.05.

Patients with peritoneal endometriosis or deep infiltrating endometriosis of uterosacral ligament were divided into three groups: mild pain group, moderate pain group and severe pain group. We found out that as the aggravation of the grade of dysmenorrhea, the expression of Sema 3A in stromal cells of endometriotic lesion decreased (PEM p = 0.004; USL-EM p = 0.046); while the Anti-PGP 9.5 (+) endometriosis-associated nerve fiber density increased (PEM: p = 0.004; USL-EM p = 0.017) (Fig 6).

## Discussion

Endometriosis is an estrogen-dependent disease and is increasingly being recognized as an inflammatory condition, which affects up to 50% of women with pelvic pain [30]. Numerous studies trying to explain the relationship between the severity of pain symptoms and anatomical or biochemical characteristics of endometriotic lesions are performed, only to find weak correlation [2,31]. The importance of nervous system in regulating endometriosis-associated pain has received much attention recently. It is reported that ectopic endometriotic implants induced their own sympathetic and sensory nerve supply both in rats and in women [2–3], which is similar to that of the healthy rat uterus. The density of Anti-PGP 9.5 positive stained nerve fibers co-localized with the peritoneal endometriotic lesions is significantly higher in peritoneal lesions of patients with painful endometriosis than in patients without pain [6,27], which suggests a direct association between pain and NFD. Moreover, there is a newly recognized phenomenon that clarifies the anatomical and pathophysiological alterations in/near the endometriotic lesions. Compared to healthy tissue, sympathetic nerve fibers were found to be decreased in intestinal endometriosis [7]. And significantly reduced sympathetic innervation within the peritoneal endometriotic lesions was also found comparing to that of the para-endometriotic non-lesional tissue as well as healthy peritoneum [8]. The study also revealed similar findings in patients with peritoneal endometriosis, and for the first time, demonstrated a drastic reduction of sympathetic innervation in deep infiltrating endometriotic lesions of uterosacral ligament.

The aberrant sympathetic innervation, together with the altered or unaltered sensory nerve fibers, is believed to play a crucial role in elevated pain generation and the perpetuation of endometriotic changes [8]. The loss of sympathetic nerve fibers in inflammatory lesions is a hallmark of many acute or chronic inflammatory processes (such as colitis, arthritis, insulitis, etc.) [10,32–33]. Due to the reduced concentration of sympathetic anti-inflammatory neurotransmitters, an inflammatory promoting microenvironment is formed and triggers the following pain-generating signal pathways. This imbalanced sympathetic and sensory innervation is an evolutionarily adaptive program that is modulated by the maintenance of the functional balance of both pro-inflammatory and anti-inflammatory factors [9]. Previous studies and results of this study demonstrated alteration of sympathetic nerve fiber density is restricted to the inflamed area surrounding the endometriotic lesions, which is considered to be supportive evidence of the theory illustrated above. However, the exact mechanism underlying this interesting abnormality in endometriosis needs to be further investigated.

Because the disappearance of sympathetic nerve fibers is relatively uniform in the setting of inflammation, specific mechanisms such as nerve repellent factors have been proposed to play an important causal role [11–12]. Straub et al. had demonstrated a marked up-regulation of sympathetic nerve repellent factor (semaphorin 3F) in synovial tissue of rheumatoid arthritis [9]. Another study showed that the stronger expression of Sema 3F and Sema 3A might play an important propagating role for nerve fiber loss and contribute to the pro-proliferative and vasodilatory environment in colorectal adenomatous polyp [13]. Accordingly, we hypothesize that Sema 3A and its receptors may take part in the dysregulation of sympathetic innervation in endometriosis.

**Fig 7:**
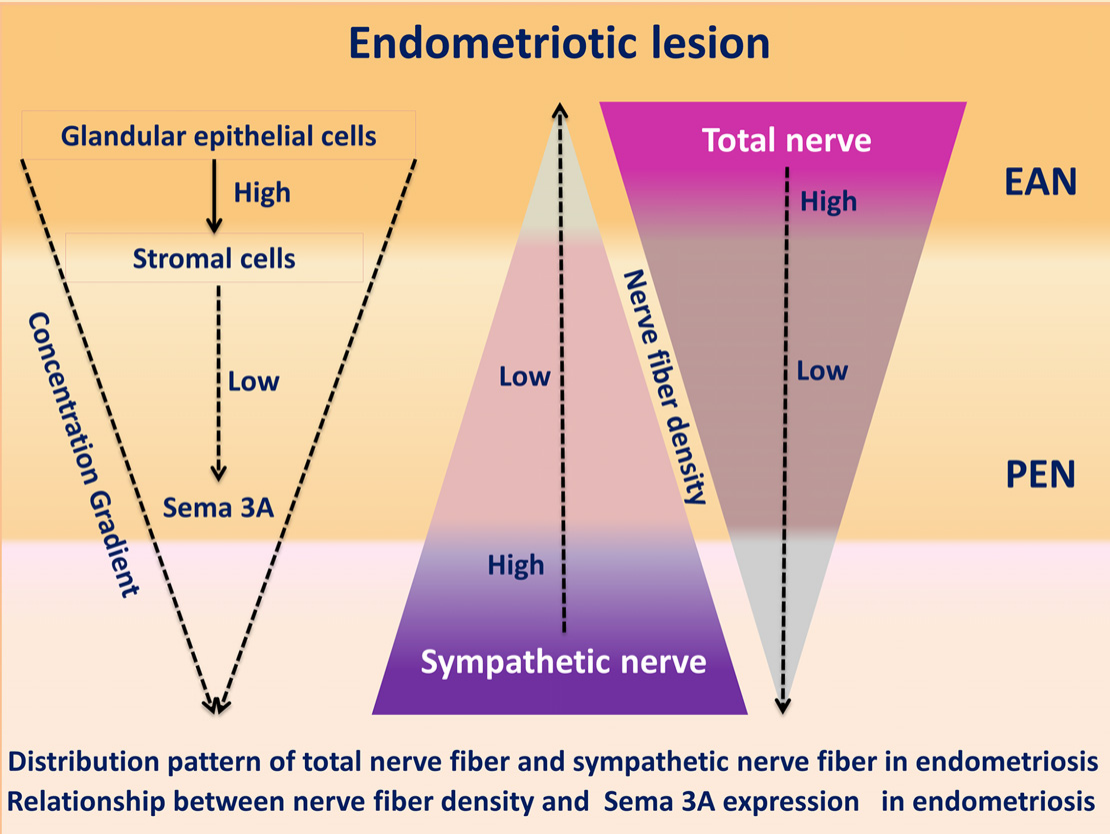
Distribution pattern of total nerve fiber and sympathetic nerve fiber in endometriosis. Relationship between nerve fiber density and Sema 3A expression in endometriosis.

To the best of our knowledge, this is the first study investigating the potential role of Sema 3A in regulating aberrant sympathetic innervation in peritoneal and deep infiltrating endometriosis (Fig 7). Kao et al. first investigated the expression of Semaphorin family in endometriosis at RNA level and found that Sema E (also named Sema 3C) was one of the up-regulated genes in eutopic endometrium from women with endometriosis, compared to that from women without endometriosis [34]. But the expression of Sema 3A in endometriosis was unknown. The expression of Sema 3A in glandular epithelial cells of peritoneal and deep infiltrating endometriotic lesions is significantly higher than that from eutopic endometrium of women with and without endometriosis. But the immunostaining of Sema 3A in ectopic stromal cells of peritoneal and deep infiltrating endometriosis is markedly reduced compared with that of the eutopic stromal cells of patients with and without endometriosis. More importantly, the expression of Sema 3A in stromal cells of endometriotic lesion is reversed related to the severity of pain as well as the total endometriosis-associated nerve fiber density (Fig 6). The Anti-PGP 9.5 (+) endometriosis-associated nerve fiber density in patients with endometriosis (either PEM or USL-EM) is significantly positively relative to the severity of dysmenorrhea, indicating a close association between totalnerve fiber density and pain. Also, the decreased expression of Sema 3A in ectopic stromal cells of patients with more severe pain suggests that Sema 3A may play a role in the formation of endometriosis-associated pain. The attenuated nerve-repelling effect of Sema 3A in endometriotic lesion results in a disinhibition of neural growth, which increases the NFD of Anti-PGP 9.5 (+) endometriosis-associated nerve. This interesting phenomenon is in consistent with the study of Tolofari et al. [23] and indicates that Sema 3A plays a potential role in regulating the imbalanced innervation of endometriosis, and it may also be involved in the formation of neuropathic pain in patients with peritoneal and deep infiltrating endometriosis.

Sema 3A is known to be a neuronal chemorepellent acting as an important axonal guidance molecule during development of both central nervous system (CNS) and peripheral nervous system (PNS). The pattern of Sema 3A expression by neurons is altered upon specific pathophysiologic conditions (inflammation, injury, etc.), thus leading to differing levels of axonal regeneration in CNS and PNS [35–37]. It facilitates the repulsion of sensory and sympathetic axonal growth cones [38–39]. NRP-1 is a transmembrane receptor of Sema 3A. After forming receptor complexes with Plexins, NRP-1 propagates Sema 3A signals into neurons and initiates the downstream activation of distinct cyclic nucleotide signalling pathways (cAMP and cGMP) [39]. Other than its neuronal repulsive function, Sema 3A has been also shown to have diverse and important functions in other physiological and pathological processes, including organogenesis, vasculogenesis, immune cell regulation, and tumor progression [40–42].

Higher expression of Sema 3A in eutopic glandular epithelial cells of women with endometriosis than that from women without endometriosis indicates the notion that significant alterations occur in the intrauterine environment of patients with endometriosis. Furthermore, the expression of Sema 3A is markedly enhanced in ectopic glandular epithelial cells of peritoneal and deep infiltrating endometriotic lesions. This up-regulation of Sema 3A is proposed to be one of the specific characteristics induced by multiple factors in the pathogenesis of endometriosis. More importantly, the NFD of sympathetic nerve within the lesion was significantly reduced, accompanying with the high level of Sema 3A in ectopic glandular epithelial cells and low level of Sema 3A in ectopic stromal cells. These results suggest that Sema 3A is a potential molecule regulating the aberrant sympathetic innervation in peritoneal and deep infiltrating endometriosis. There are several possible mechanisms of action underlying this interesting neuromodulatory process.

The NFDs of Anti-PGP 9.5 (+) endometriosis-associated nerve (either peritoneal endometriosis or deep infiltrating endometriosis of uterosacral ligament) are higher than NFDs of para-endometriotic nerve and nerve of healthy tissues (either healthy peritoneum or healthy uterosacral ligament). But the NFD of endometriosis-associated sympathetic nerve is inversely decreased. This observation raises the possibility that functionally distinct types of pelvic nerves (e.g. sensory or sympathetic nerves) may have different exposure or responses to Sema 3A [39]. On one hand, the increased expression of Sema 3A in ectopic glandular epithelial cells may be part of repair or rescue mechanism to prevent neural ingrowth into the endometriotic lesions [23]. We propose that there exists a negative feedback loop between the epithelial cells and stromal cells. The reduced expression of Sema 3A in stromal cells reversely activates the synthesis and secretion of Sema 3A in the epithelial cells, thus compensating the attenuate neuronal repulsive effect in the lesions. On the other hand, the response of sensory nerve fibers to the axonal repulsive effect of Sema 3A in the ectopic glandular epithelial cells may be weaker than the response of sympathetic nerves to Sema 3A. Besides, there are many other nerve growth promoters within the endometriotic lesions or the peritoneal fluid. The promoting effect of axonal growth factors to sensory nerves is much stronger than the inhibitory effect exerted by Sema 3A, whereas sympathetic nerves do have weak response to these factors [43]. Consequently, the increase of sensory nerve fibers counteracts or even exceeds the loss of sympathetic nerve fibers within the endometriotic lesions. Finally, the NFD of endometriosis-associated total nerve fibers are greater than that of the para-endometriotic nerve fibers.

**Fig 8:**
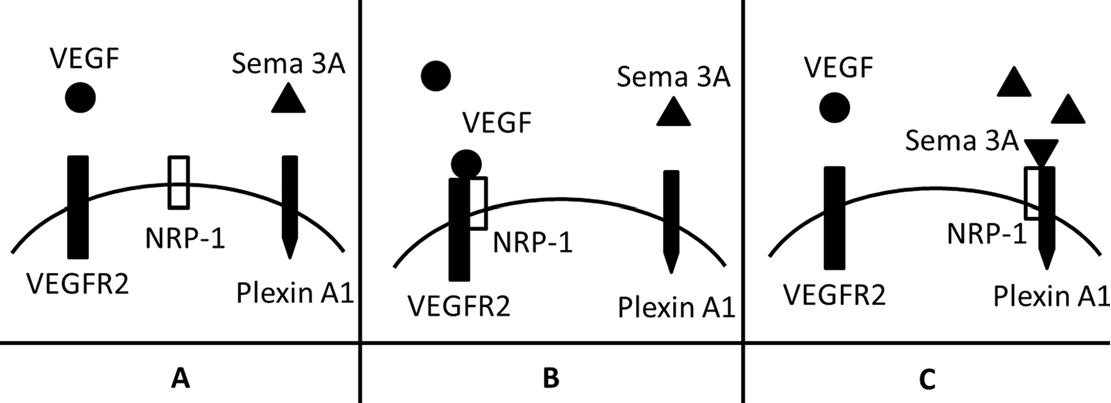
Modulation mechanisms of sympathetic innervation by different levels of Sema 3A. (A) NPR-1 is a co-receptor of VEGFR2, which is involved in VEGF signaling; NRP-1 is also a receptor for Sema 3A, after triggered by Sema 3A, it binds to Plexin A1, forming a complex implicated in modulating sympathetic innervation. (B) When the concentration of Sema 3A is low, VEGF-NRP-1-VEGFR2 signaling is dominant, thus providing chemoattractive cues for sympathetic nerve outgrowth. (C) When Sema 3A is high expressed, it mainly triggers NPR-1-VEGFR2 signaling, producing chemorepulsive cues limiting sympathetic nerve outgrowth.

When Sema 3A is secreted from the endometriotic glandular epithelial and stromal cells, it forms a concentration gradient from the highest point within the lesion to the lowest point near the lesion (para-endometriotic tissues) [43]. Sema 3A acts as a stop signal for the growth of sympathetic axons and arrests them at the bottom of the Sema 3A gradient. The result of this concentration-dependent manner of NRP-1 mediated Sema 3A signals [40,44] is the accumulation of sympathetic neurons in the para-endometriotic tissues (Fig 7). High levels of Sema 3A favor NPR-1-Plexin A1 signaling, which produced chemorepulsive cues limiting sympathetic neurite (Fig 8C); while low Sema 3A expression favors NPR-1-VEGFR2 signaling, providing chemoattractive cues for sympathetic neurite outgrowth [25] (Fig 8B). Another possible mechanism is that there is a reverse stimulation to the remaining axons induced by the repulsion of target sympathetic axons itself [9]. The cytoplasm, as well as cell organelles and neurotransmitter-loaded vesicles of the repelled sympathetic axon will be taken up by the remaining shortened axon or neuronal soma. As a result, the remaining axons in the para-endometriotic tissues will be well nourished and generate more spiral or coiled thickened axon bundles.

## Conclusion

In conclusion, the results obtained in the present study suggest that Sema 3A and its receptors play an important potential role of regulating the aberrant sympathetic innervation of peritoneal and deep infiltrating endometriosis. The identification of sympathetic axonal repulsive effect induced by Sema 3A provides a new insight of nerve repellent factors in modulating the abnormal innervation of endometriotic lesions.

## Supporting Information

S1 Table HSCORE of Sema 3A, Plexin A1 and NRP-1 of glandular epithelial cells as well as stromal cells from eutopic endometrium from patients with endometriosis (EM) and without endometriosis (NEM). (DOCX)

S2 Table HSCORE of Sema 3A, Plexin A1 and NRP-1 of glandular epithelial cells as well as stromal cells from eutopic endometrium of different phase of the menstrual cycle. (DOCX)

S3 Table Comparison of total nerve fiber density (NFD, NF/mm^2^) in peritoneal endometriotic specimens and healthy peritoneum. EAN-PEM: endometriosis-associated nerve of peritoneal endometriosis; PEN-PEM: para-endometriotic nerve of peritoneal endometriosis; N-PC: nerve of peritoneum of control.
(DOCX)

S4 Table Comparison of sympathetic nerve fiber density (NFD, NF/mm^2^) in peritoneal endometriotic specimens and healthy peritoneum. ESAN-PEM: Endometriosis-associated sympathetic nerve of peritoneal endometriosis; PESN-PEM: Para-endometriotic sympathetic nerve of peritoneal endometriosis; SN-PC: sympathetic nerve of peritoneum of control.
(DOCX)

S5 Table Comparison of total nerve fiber density (NFD, NF/mm^2^) in deep infiltrating endometriotic specimens of uterosacral ligament and healthy uterosacral ligament. EAN-USL-EM: endometriosis-associated nerve of deep infiltrating endometriosis of uterosacral ligament; PEN-USL-EM: para-endometriotic nerve of deep infiltrating endometriosis of uterosacral ligament; N-USL-C: nerve of uterosacral ligament of control.
(DOCX)

S6 Table Comparison of sympathetic nerve fiber density (NFD, NF/mm^2^) in deep infiltrating endometriotic specimens of uterosacral ligament and healthy uterosacral ligament. ESAN-USL-EM: endometriosis-associated sympathetic nerve of USL-EM, PESN-USL-EM: para-endometriotic sympatheticnerve of USL-EM; SN-USL-C: sympathetic NFD of uterosacral ligament of control.
(DOCX)

S1 Fig Positive control of Sema 3A. **Sema 3A was positively stained in glands of intestinal mucosa.** (immunohistochemical stain, 100 × magnification)
(DOCX)

S2 Fig Positive control of Plexin A1. **Plexin A1 was positively stained in glandular cells of rectal mucosa.** (immunohistochemical stain, 100 × magnification)
(DOCX)

S3 Fig Positive control of NRP-1. **NRP-1 was positively stained in nerve fibers.** (immunohistochemical stain, 100 × magnification)
(DOCX)

S4 Fig The expression of Sema 3A (white arrow) in stromal cells of eutopic endometrium from patients with endometriosis. (immunohistochemical stain, 200 × magnification)
(DOCX)

S5 Fig The expression of Sema 3A (white arrow) in stromal cells of eutopic endometrium from patients without endometriosis. (immunohistochemical stain, 200 × magnification)
(DOCX)

S6 Fig The expression of Plexin A1 (white arrow) in stromal cells of eutopic endometrium from patients with endometriosis. (immunohistochemical stain, 200 × magnification)
(DOCX)

S7 Fig The expression of Plexin A1 (white arrow) in stromal cells of eutopic endometrium from patients without endometriosis. (immunohistochemical stain, 200 × magnification)
(DOCX)

S8 Fig The expression of Sema 3A (white arrow) in stromal cells of ectopic endometrium from patients with deep infiltrating endometriosis of uterosacral ligament. (immunohistochemical stain, 200 × magnification)
(DOCX)

S9 Fig The expression of Sema 3A (white arrow) in stromal cells of ectopic endometrium from patients with pelvic endometriosis. (immunohistochemical stain, 200 × magnification)
(DOCX)

S10 Fig The expression of Plexin A1 (white arrow) in stromal cells of ectopic endometrium from patients with deep infiltrating endometriosis of uterosacral ligament. (immunohistochemical stain, 200 × magnification)
(DOCX)

S11 Fig The expression of Plexin A1 (white arrow) in stromal cells of ectopic endometrium from patients with pelvic endometriosis. (immunohistochemical stain, 200 × magnification)
(DOCX)

S12 Fig Immunohistochemical staining of Sema 3A in glandular epithelial cells of the eutopic endometrium from women with endometriosis (B) or without endometriosis (A), and glandular epithelial cells of PEM (C) and USL-EM (D). PEM: peritoneal endometriosis; USL-EM: deep infiltrating endometriosis of uterosacral ligament. (immunohistochemical stain, 200 × magnification)
(DOCX)

S13 Fig Immunofluorescence histochemical double staining of Sema 3A and target nerve fibers. A: Merged image of double staining of both Sema 3A and TH (white arrow: peritoneal endometriotic glands, Sema 3A positive stained in green; white triangle: endometriosis-associated sympathetic nerve, TH positive stained in orange yellow; nuclei were stained in blue by DAPI staining). B: Merged image of double staining of both Sema 3A and PGP 9.5 (white arrow: endometriotic glands of deep infiltrating endometriosis of uterosacral ligament, Sema 3A positive stained in green; white triangle: PGP 9.5 positive stained endometriosis-associated nerve, orange yellow; nuclei were stained in blue by DAPI staining). Original magnification: 200×. TH: Tyrosine hydroxylase.
(DOCX)
