# Supplementary material for: Potential Role of Semaphorin 3A and Its Receptors in Regulating Aberrant Sympathetic Innervation in Peritoneal and Deep Infiltrating Endometriosis
Source: PLoS One. 2015 Dec 31;10(12):e0146027. doi: 10.1371/journal.pone.0146027 (PMC4697795; doi:10.1371/journal.pone.0146027)
Supplement: S1 Table — (DOCX) [file pone.0146027.s001.docx]

**S1 Table HSCORE of Sema 3A, Plexin A1 and NRP-1 of glandular epithelial cells as well as stromal cells from eutopic endometrium from patients with endometriosis (EM) and without endometriosis (NEM)**

| Cells (Group) | n | HSCORE(‾x±s) | | |
| --- | --- | --- | --- | --- |
|  |  | Sema 3A | Plexin A1 | NRP-1 |
| Glandular epithelial cells (EM) | 22 | 309.09±66.61 | 288.64±40.63 | 256.82±35.51 |
| Glandular epithelial cells (NEM) | 26 | 271.15±49.34 | 257.69±48.36 | 238.46±38.23 |
| Stromal cells (EM) | 22 | 236.36±49.24 | 268.18±39.48 | 334.09±32.32 |
| Stromal cells (NEM) | 26 | 242.31±48.36 | 255.77±43.19 | 300.0±40.00 |
