# Supplementary material for: Potential Role of Semaphorin 3A and Its Receptors in Regulating Aberrant Sympathetic Innervation in Peritoneal and Deep Infiltrating Endometriosis
Source: PLoS One. 2015 Dec 31;10(12):e0146027. doi: 10.1371/journal.pone.0146027 (PMC4697795; doi:10.1371/journal.pone.0146027)
Supplement: S2 Table — (DOCX) [file pone.0146027.s002.docx]

**S2 Table HSCORE of Sema 3A, Plexin A1 and NRP-1 of glandular epithelial cells as well as stromal cells from eutopic endometrium of different phase of the menstrual cycle.**

**Group 1 Patients without endometriosis (NEM)**

| Cells(NEM) | Phase | n | HSCORE(‾x±s) | | |
| --- | --- | --- | --- | --- | --- |
|  |  |  | Sema 3A | Plexin A1 | NRP-1 |
| Glandular epithelial cells | proliferative | 14 | 282.22±50.49 | 268.69±45.22 | 229.97±39.11 |
|  | secretory | 12 | 267.33±46.21 | 245.11±43.26 | 250.09±36.65 |
| Stromal cells | proliferative | 14 | 249.76±40.28 | 261.86±40.10 | 293.02±44.13 |
|  | secretory | 12 | 239.55±49.54 | 250.29±43.98 | 307.37±43.06 |

**Group 2 Patients with endometriosis (EM)**

| Cells(EM) | Phase | n | HSCORE(‾x±s) | | |
| --- | --- | --- | --- | --- | --- |
|  |  |  | Sema 3A | Plexin A1 | NRP-1 |
| Glandular epithelial cells | proliferative | 14 | 292.23±45.27 | 297.44±47.21 | 244.11±32.01 |
|  | secretory | 8 | 320.62±50.45 | 273.36±40.18 | 266.76±34.92 |
| Stromal cells | proliferative | 14 | 221.95±40.55 | 274.23±41.21 | 325.18±35.26 |
|  | secretory | 8 | 245.75±46.47 | 258.14±46.27 | 346.07±41.76 |
