# Supplementary material for: Potential Role of Semaphorin 3A and Its Receptors in Regulating Aberrant Sympathetic Innervation in Peritoneal and Deep Infiltrating Endometriosis
Source: PLoS One. 2015 Dec 31;10(12):e0146027. doi: 10.1371/journal.pone.0146027 (PMC4697795; doi:10.1371/journal.pone.0146027)
Supplement: S3 Table — EAN-PEM: endometriosis-associated nerve of peritoneal endometriosis; PEN-PEM: para-endometriotic nerve of peritoneal endometriosis; N-PC: nerve of peritoneum of control. (DOCX) [file pone.0146027.s003.docx]

**S3 Table Comparison of total nerve fiber density (NFD, NF/mm^2^) in peritoneal endometriotic specimens and healthy peritoneum**

| Group | n | total NFD (‾x±s, NF/mm^2^**)** |
| --- | --- | --- |
| EAN-PEM | 24 | 7.54±4.85 |
| PEN-PEM | 24 | 1.61±0.98 |
| N-PC | 13 | 2.98±2.22 |

EAN-PEM: endometriosis-associated nerve of peritoneal endometriosis; PEN-PEM: para-endometriotic nerve of peritoneal endometriosis; N-PC: nerve of peritoneum of control.
