# Supplementary material for: Potential Role of Semaphorin 3A and Its Receptors in Regulating Aberrant Sympathetic Innervation in Peritoneal and Deep Infiltrating Endometriosis
Source: PLoS One. 2015 Dec 31;10(12):e0146027. doi: 10.1371/journal.pone.0146027 (PMC4697795; doi:10.1371/journal.pone.0146027)
Supplement: S4 Table — ESAN-PEM: Endometriosis-associated sympathetic nerve of peritoneal endometriosis; PESN-PEM: Para-endometriotic sympathetic nerve of peritoneal endometriosis; SN-PC: sympathetic nerve of peritoneum of control. (DOCX) [file pone.0146027.s004.docx]

**S4 Table Comparison of sympathetic nerve fiber density (NFD, NF/mm^2^) in peritoneal endometriotic specimens and healthy peritoneum**

| Group | n | sympathetic NFD (‾x±s, NF/mm^2^**)** |
| --- | --- | --- |
| EASN-PEM | 24 | 0.70±0.36 |
| PESN-PEM | 24 | 1.12±0.62 |
| SN-PC | 13 | 1.71±0.86 |

ESAN-PEM: Endometriosis-associated sympathetic nerve of peritoneal endometriosis; PESN-PEM: Para-endometriotic sympathetic nerve of peritoneal endometriosis; SN-PC: sympathetic nerve of peritoneum of control.
