# Supplementary material for: Potential Role of Semaphorin 3A and Its Receptors in Regulating Aberrant Sympathetic Innervation in Peritoneal and Deep Infiltrating Endometriosis
Source: PLoS One. 2015 Dec 31;10(12):e0146027. doi: 10.1371/journal.pone.0146027 (PMC4697795; doi:10.1371/journal.pone.0146027)
Supplement: S5 Table — EAN-USL-EM: endometriosis-associated nerve of deep infiltrating endometriosis of uterosacral ligament; PEN-USL-EM: para-endometriotic nerve of deep infiltrating endometriosis of uterosacral ligament; N-USL-C: nerve of uterosacral ligament of control. (DOCX) [file pone.0146027.s005.docx]

**S5 Table Comparison of total nerve fiber density (NFD, NF/mm^2^) in deep infiltrating endometriotic specimens of uterosacral ligament and healthy uterosacral ligament**

| Group | n | total NFD (‾x±s, NF/mm^2^**)** |
| --- | --- | --- |
| EAN-USL-EM | 20 | 30.07±9.55 |
| PEN- USL-EM | 20 | 9.22±2.89 |
| N- USL-C | 13 | 10.23±4.08 |

EAN-USL-EM: endometriosis-associated nerve of deep infiltrating endometriosis of uterosacral ligament; PEN-USL-EM: para-endometriotic nerve of deep infiltrating endometriosis of uterosacral ligament; N-USL-C: nerve of uterosacral ligament of control.
