# Supplementary material for: Potential Role of Semaphorin 3A and Its Receptors in Regulating Aberrant Sympathetic Innervation in Peritoneal and Deep Infiltrating Endometriosis
Source: PLoS One. 2015 Dec 31;10(12):e0146027. doi: 10.1371/journal.pone.0146027 (PMC4697795; doi:10.1371/journal.pone.0146027)
Supplement: S6 Table — ESAN-USL-EM: endometriosis-associated sympathetic nerve of USL-EM, PESN-USL-EM: para-endometriotic sympatheticnerve of USL-EM; SN-USL-C: sympathetic NFD of uterosacral ligament of control. (DOCX) [file pone.0146027.s006.docx]

**S6 Table Comparison of sympathetic nerve fiber density (NFD, NF/mm^2^) in deep infiltrating endometriotic specimens of uterosacral ligament and healthy uterosacral ligament**

| Group | n | sympathetic NFD (‾x±s, NF/mm^2^**)** |
| --- | --- | --- |
| EASN- USL-EM | 20 | 0.86±0.46 |
| PESN- USL-EM | 20 | 2.84±1.75 |
| SN- USL-C | 13 | 3.93±1.52 |

ESAN-USL-EM: endometriosis-associated sympathetic nerve of USL-EM, PESN-USL-EM: para-endometriotic sympatheticnerve of USL-EM; SN-USL-C: sympathetic NFD of uterosacral ligament of control.
