# Supplementary figures and images for: Potential Role of Semaphorin 3A and Its Receptors in Regulating Aberrant Sympathetic Innervation in Peritoneal and Deep Infiltrating Endometriosis
Source: PLoS One. 2015 Dec 31;10(12):e0146027. doi: 10.1371/journal.pone.0146027 (PMC4697795; doi:10.1371/journal.pone.0146027)

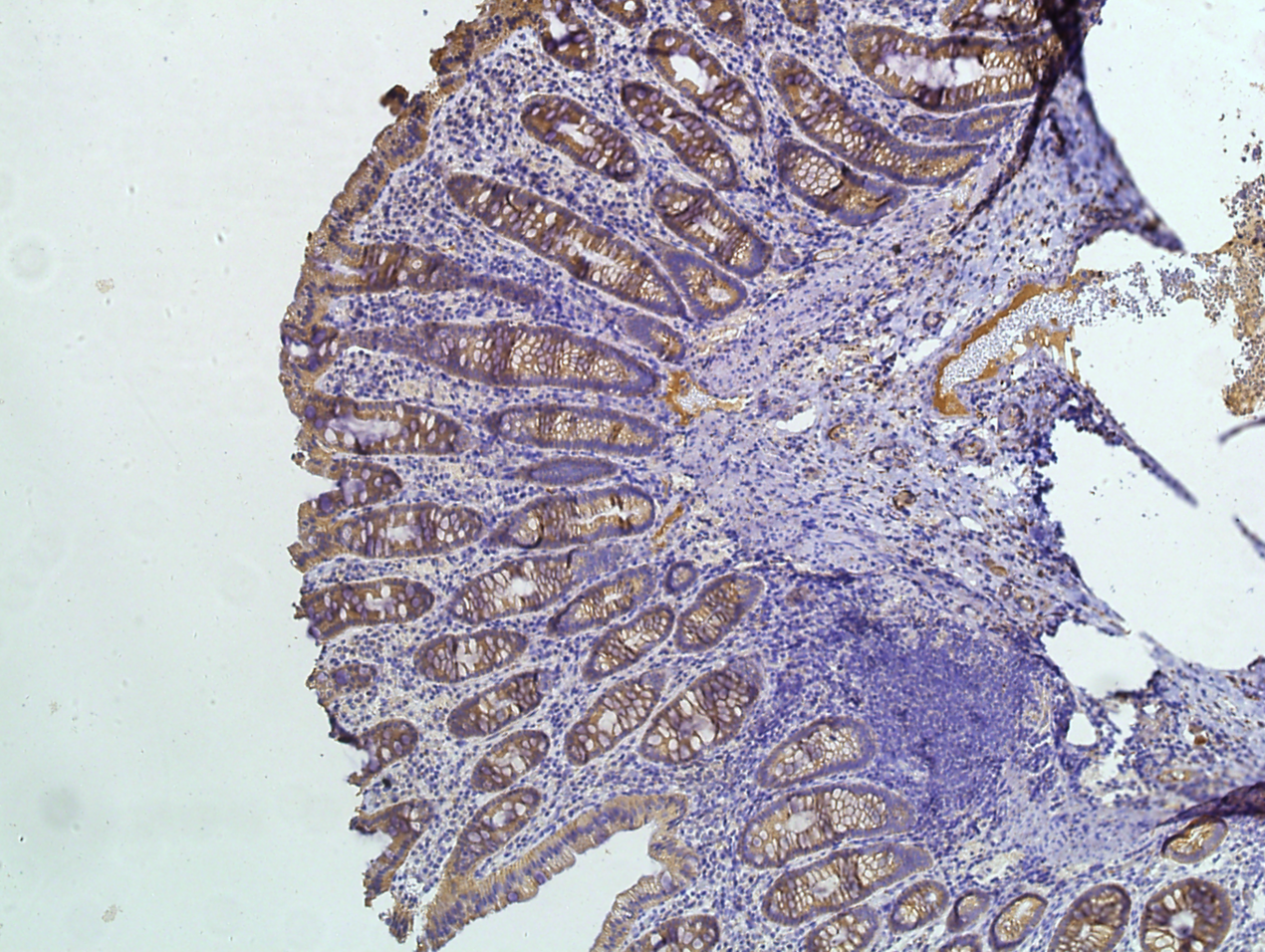

Supplement: S1 Fig — Sema 3A was positively stained in glands of intestinal mucosa. (immunohistochemical stain, 100 × magnification) (DOCX) [file pone.0146027.s007.docx]

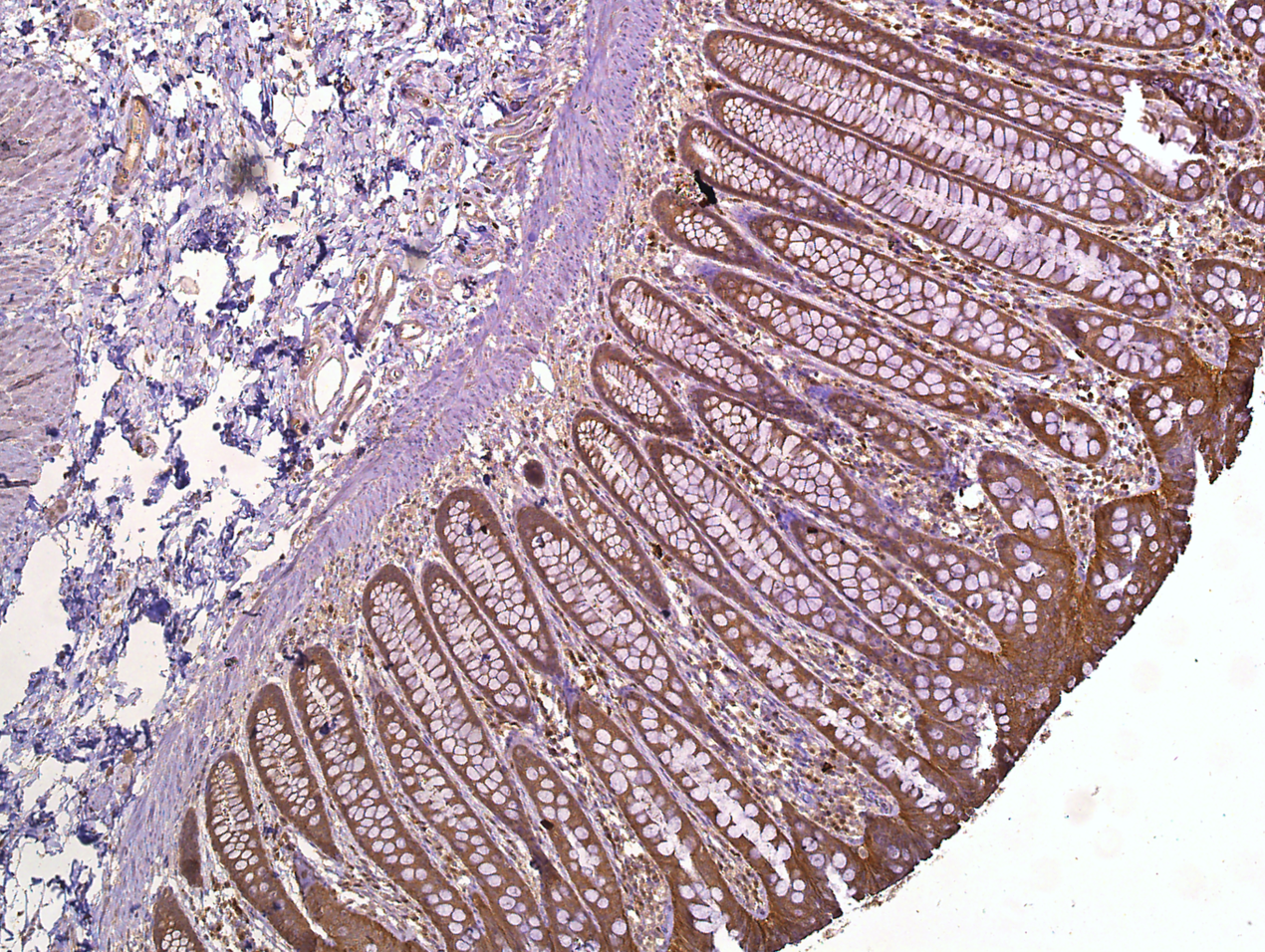

Supplement: S2 Fig — Plexin A1 was positively stained in glandular cells of rectal mucosa. (immunohistochemical stain, 100 × magnification) (DOCX) [file pone.0146027.s008.docx]

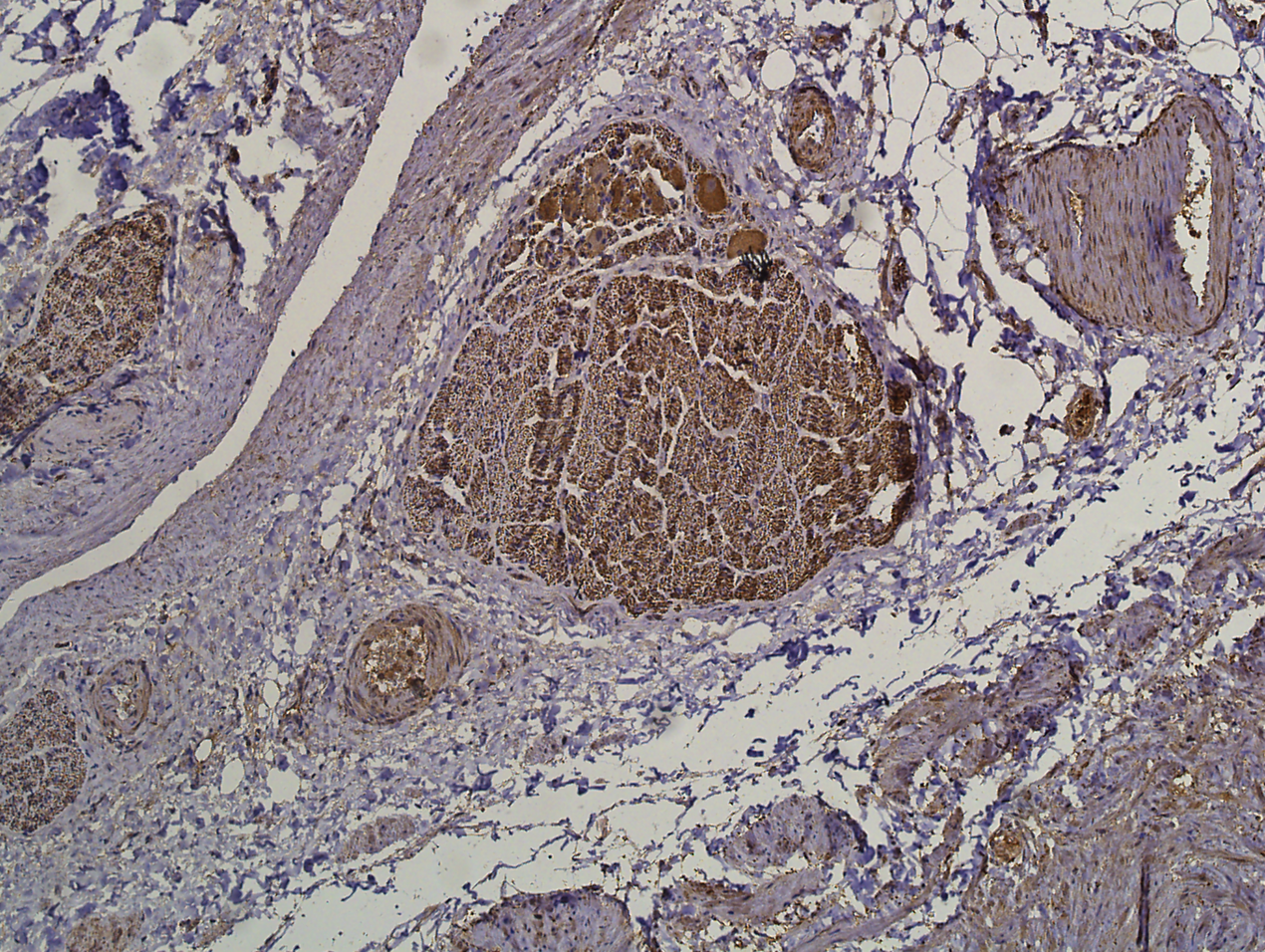

Supplement: S3 Fig — NRP-1 was positively stained in nerve fibers. (immunohistochemical stain, 100 × magnification) (DOCX) [file pone.0146027.s009.docx]

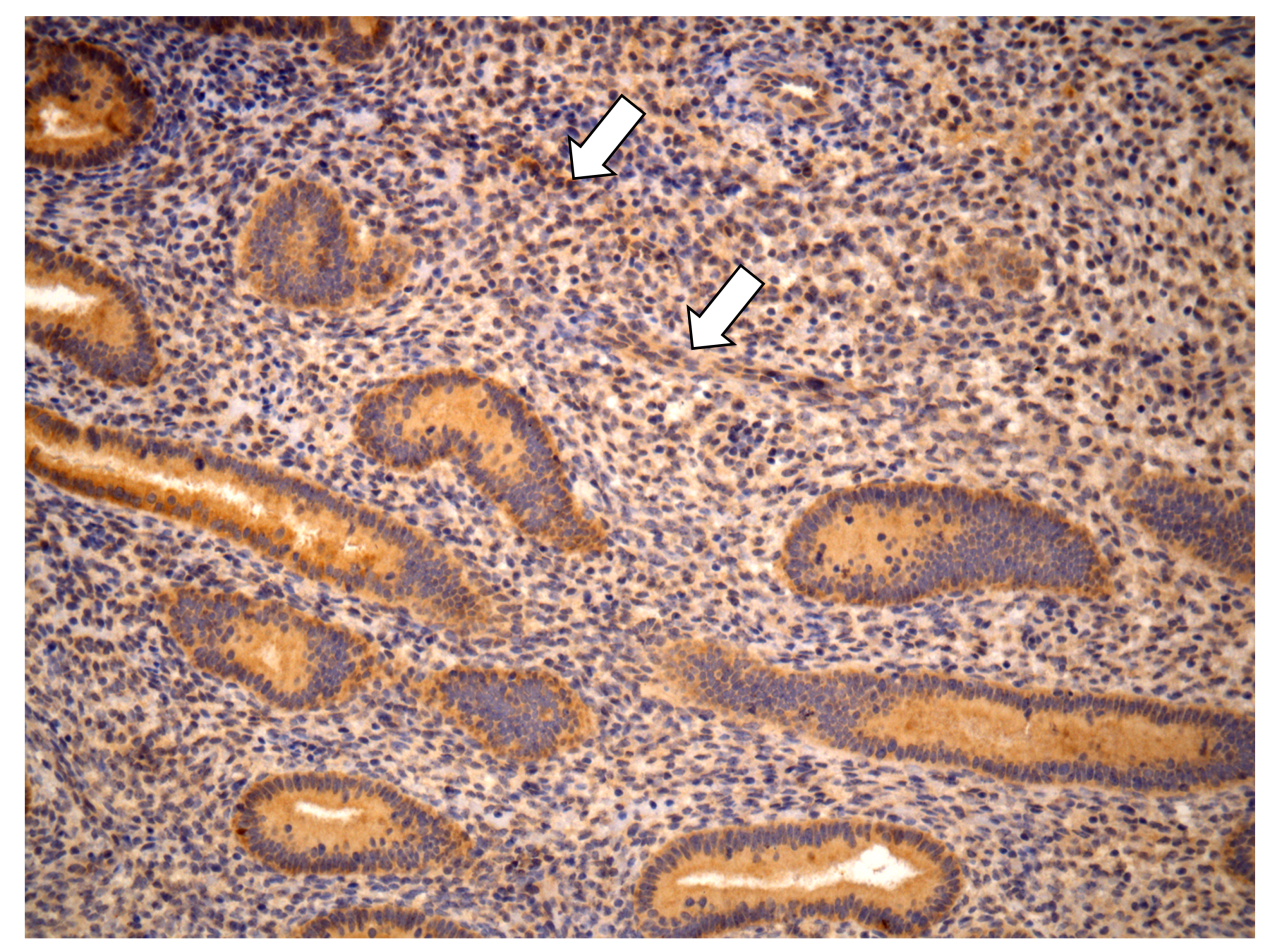

Supplement: S4 Fig — (immunohistochemical stain, 200 × magnification) (DOCX) [file pone.0146027.s010.docx]

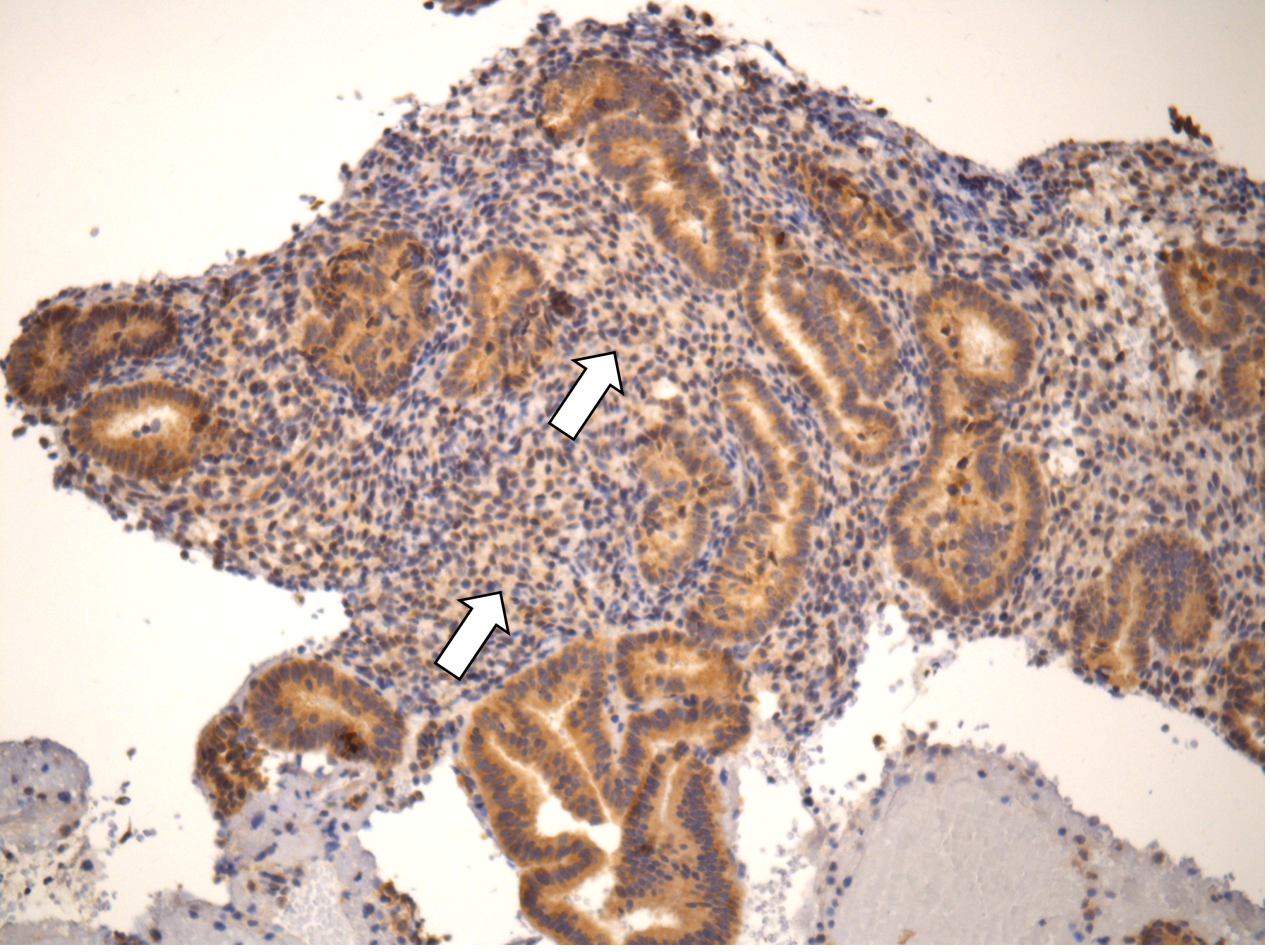

Supplement: S5 Fig — (immunohistochemical stain, 200 × magnification) (DOCX) [file pone.0146027.s011.docx]

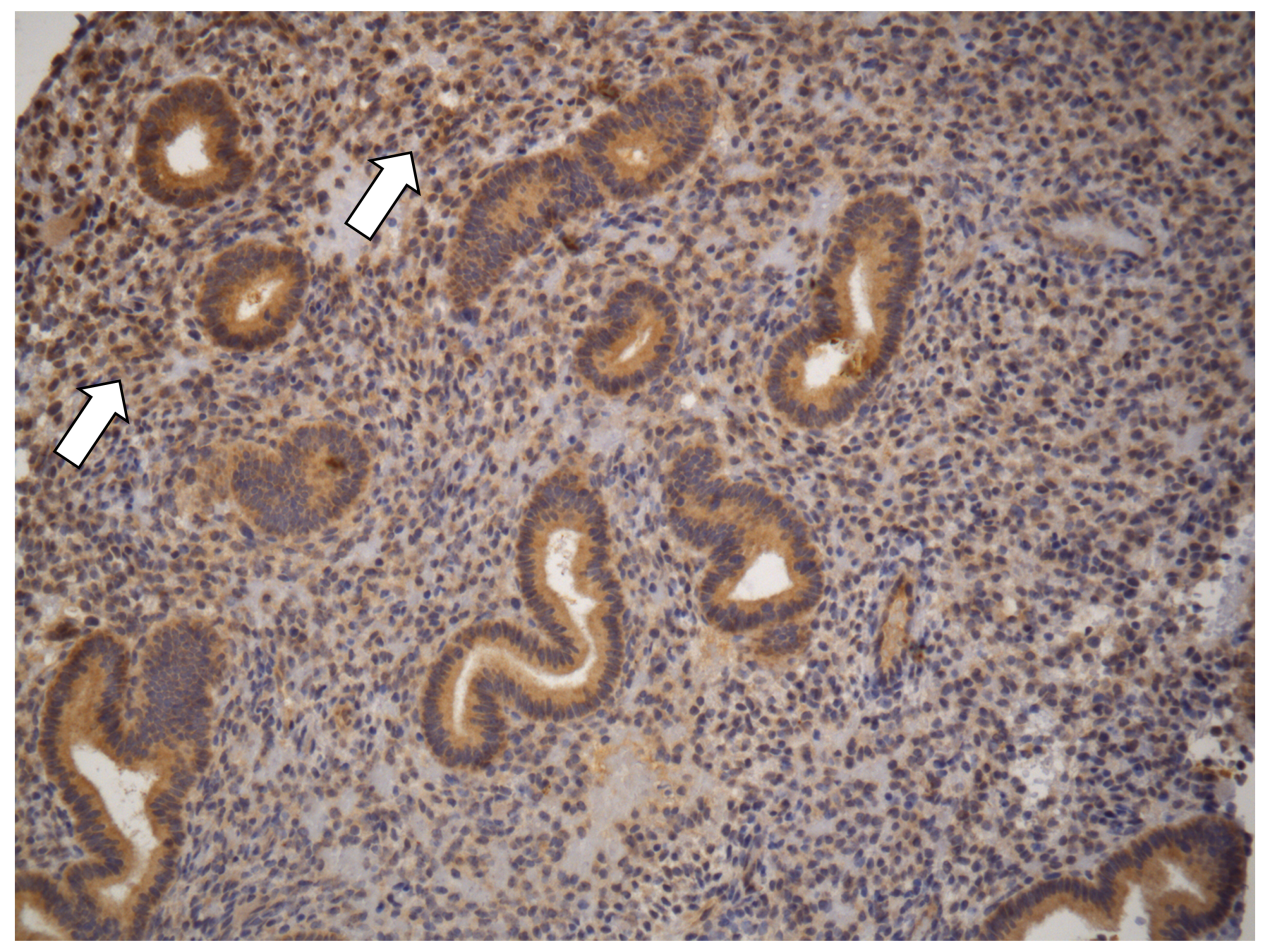

Supplement: S6 Fig — (immunohistochemical stain, 200 × magnification) (DOCX) [file pone.0146027.s012.docx]

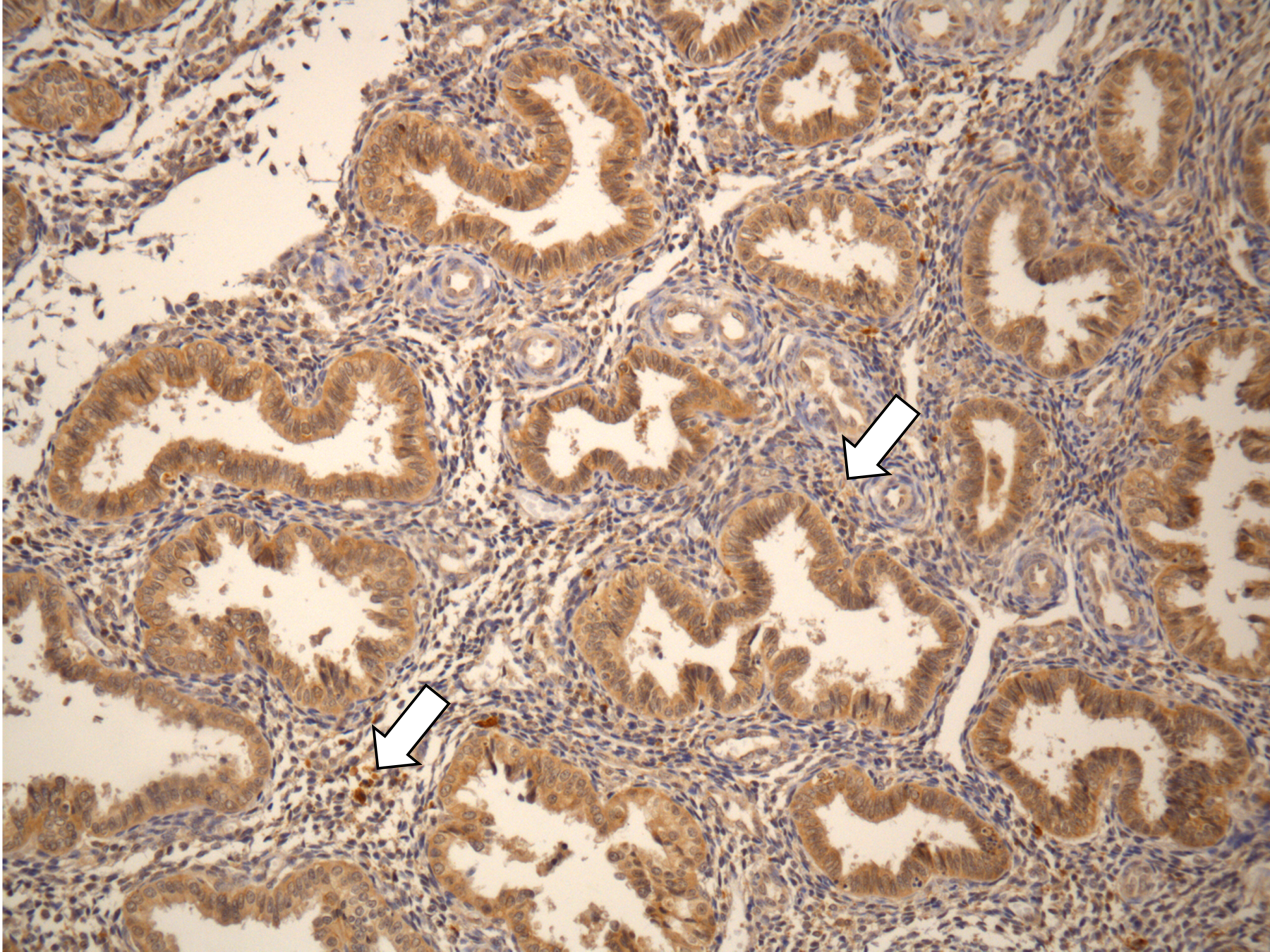

Supplement: S7 Fig — (immunohistochemical stain, 200 × magnification) (DOCX) [file pone.0146027.s013.docx]

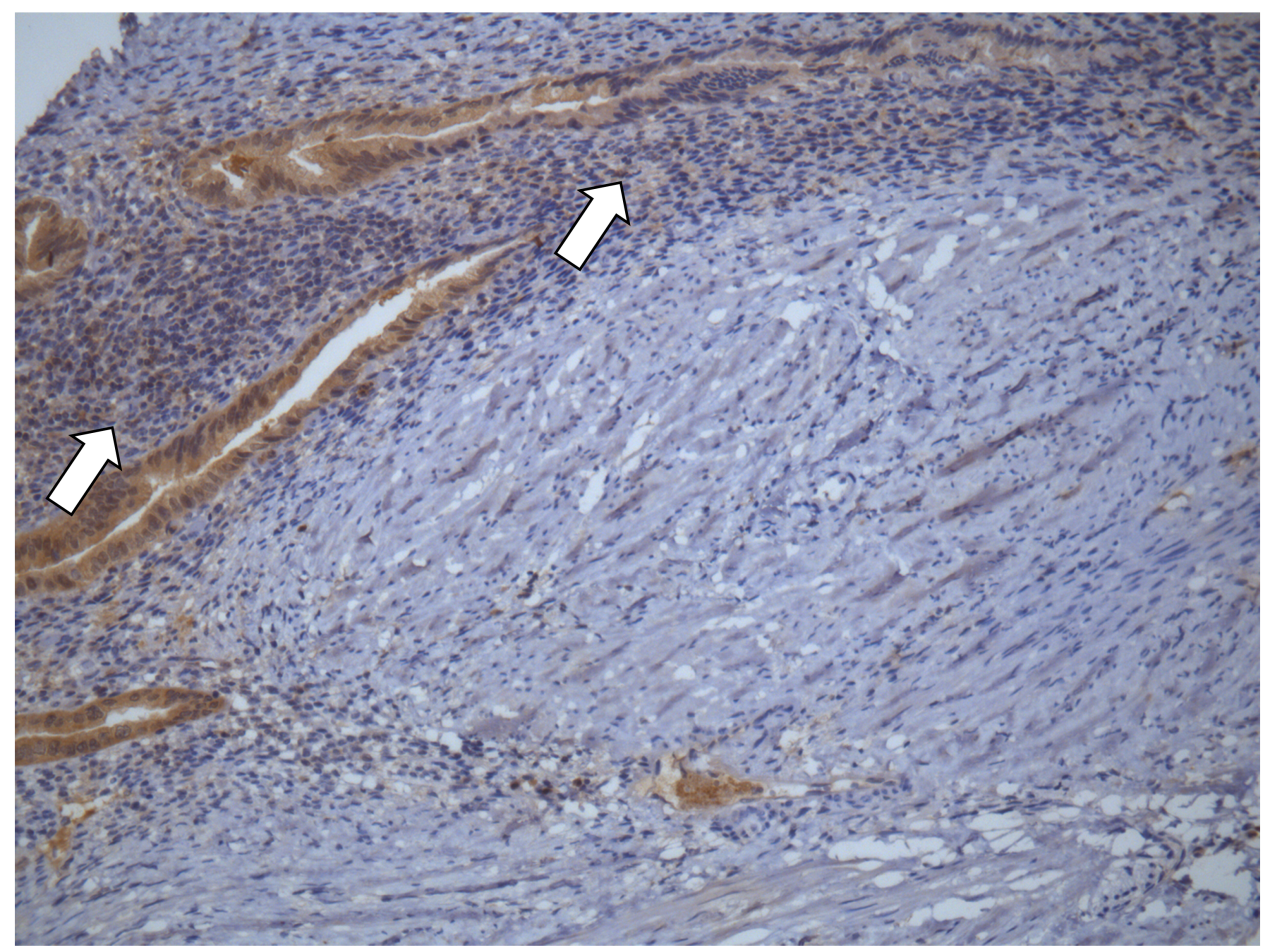

Supplement: S8 Fig — (immunohistochemical stain, 200 × magnification) (DOCX) [file pone.0146027.s014.docx]

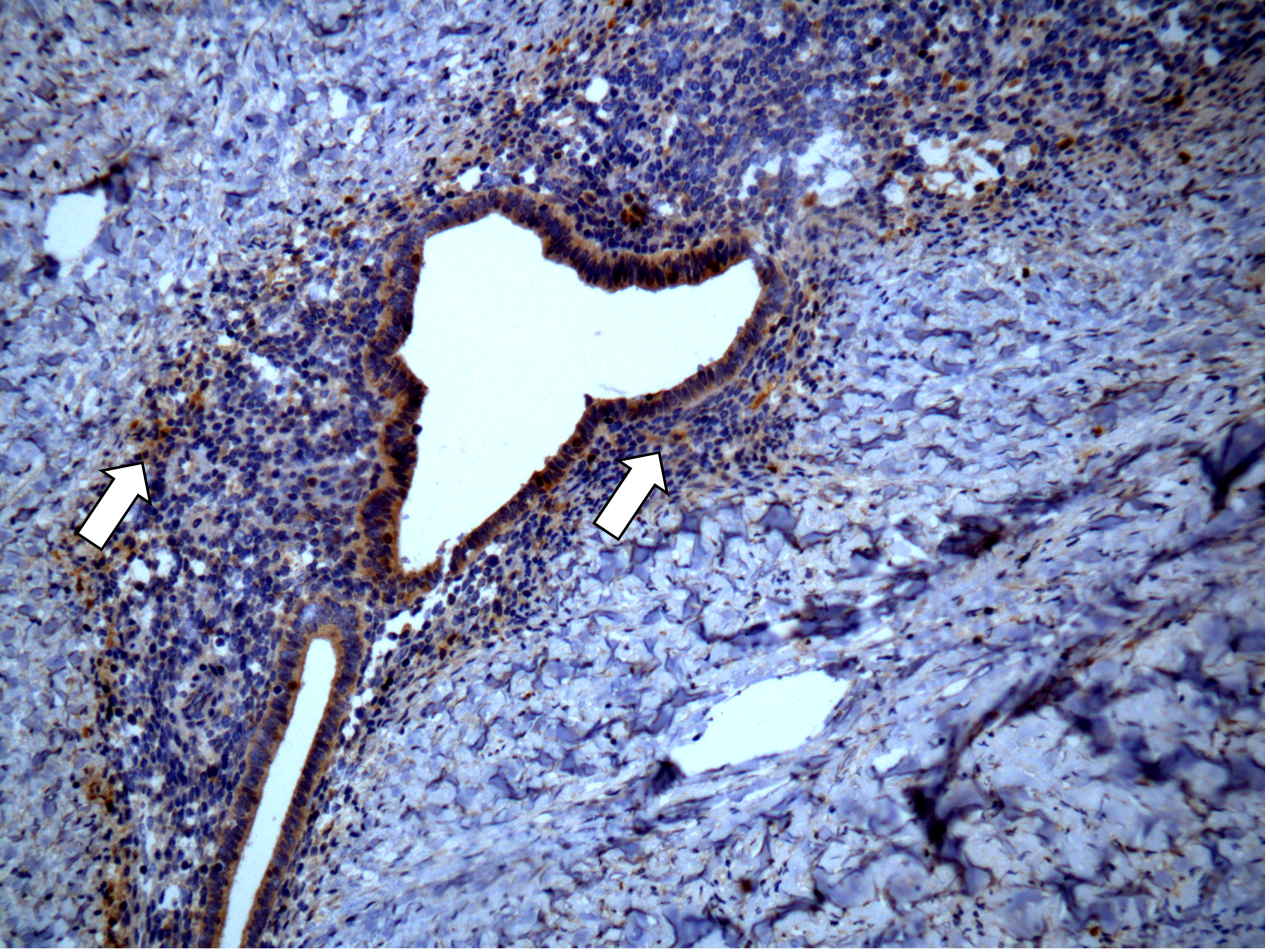

Supplement: S9 Fig — (immunohistochemical stain, 200 × magnification) (DOCX) [file pone.0146027.s015.docx]

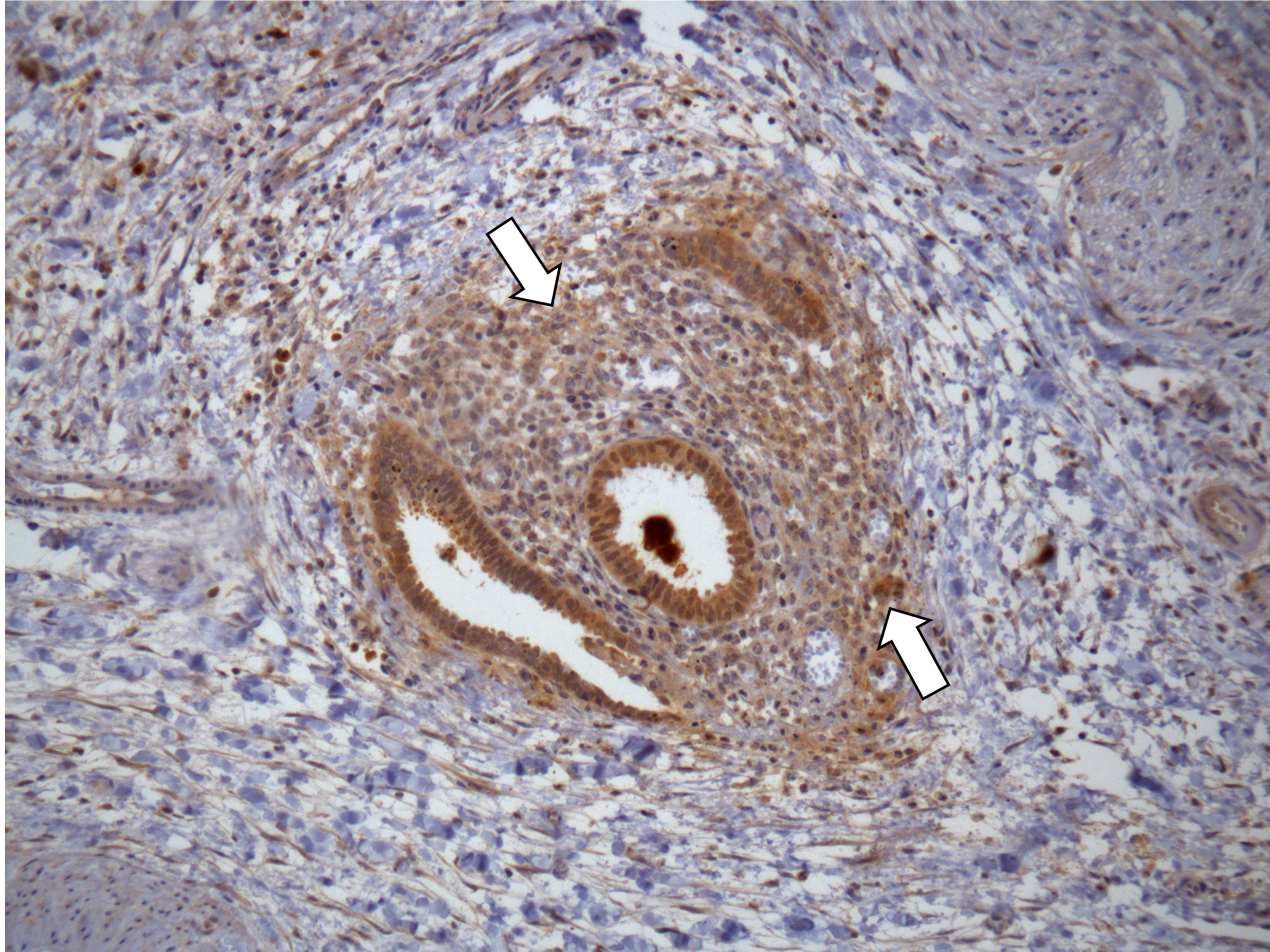

Supplement: S10 Fig — (immunohistochemical stain, 200 × magnification) (DOCX) [file pone.0146027.s016.docx]

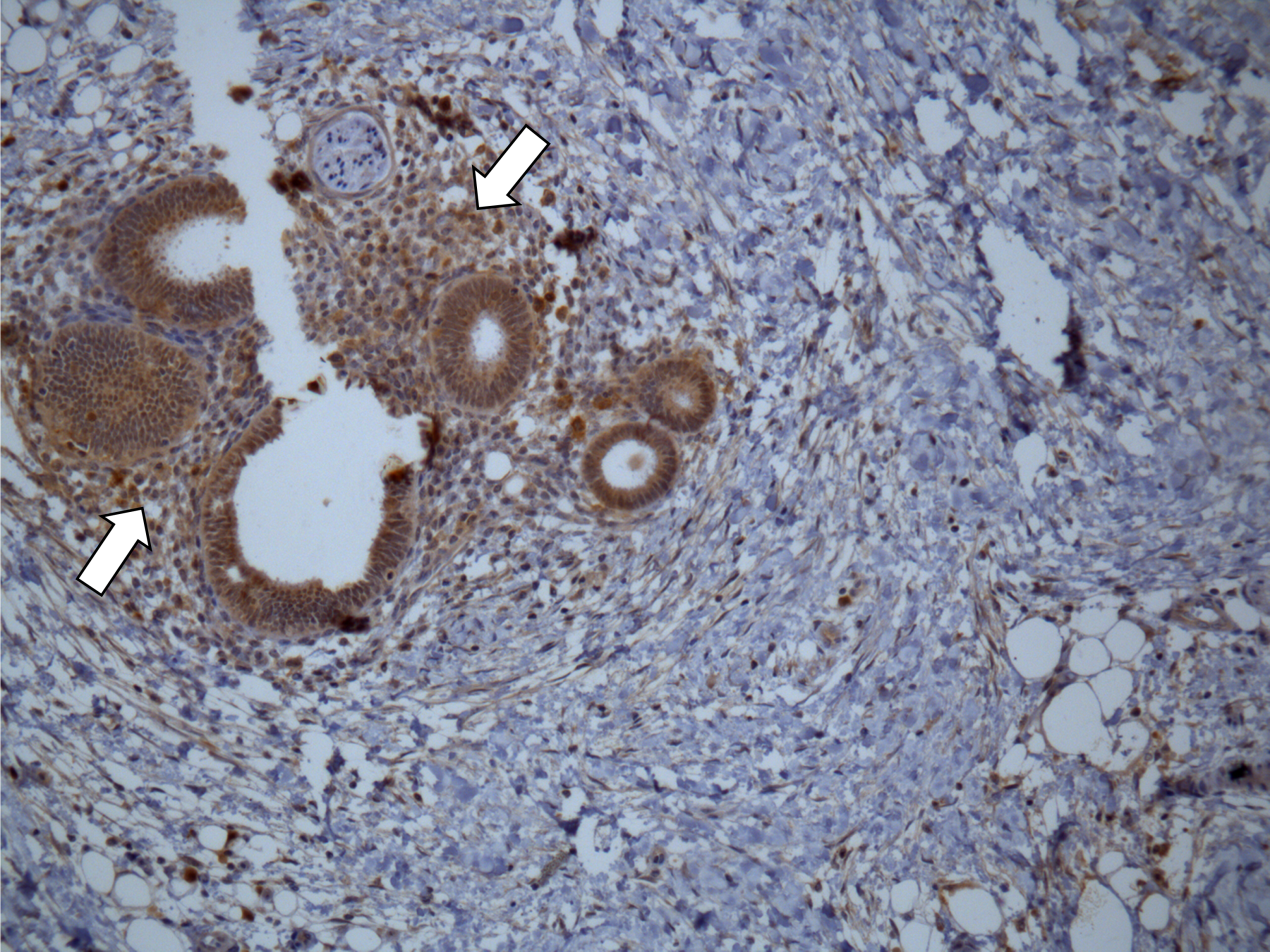

Supplement: S11 Fig — (immunohistochemical stain, 200 × magnification) (DOCX) [file pone.0146027.s017.docx]

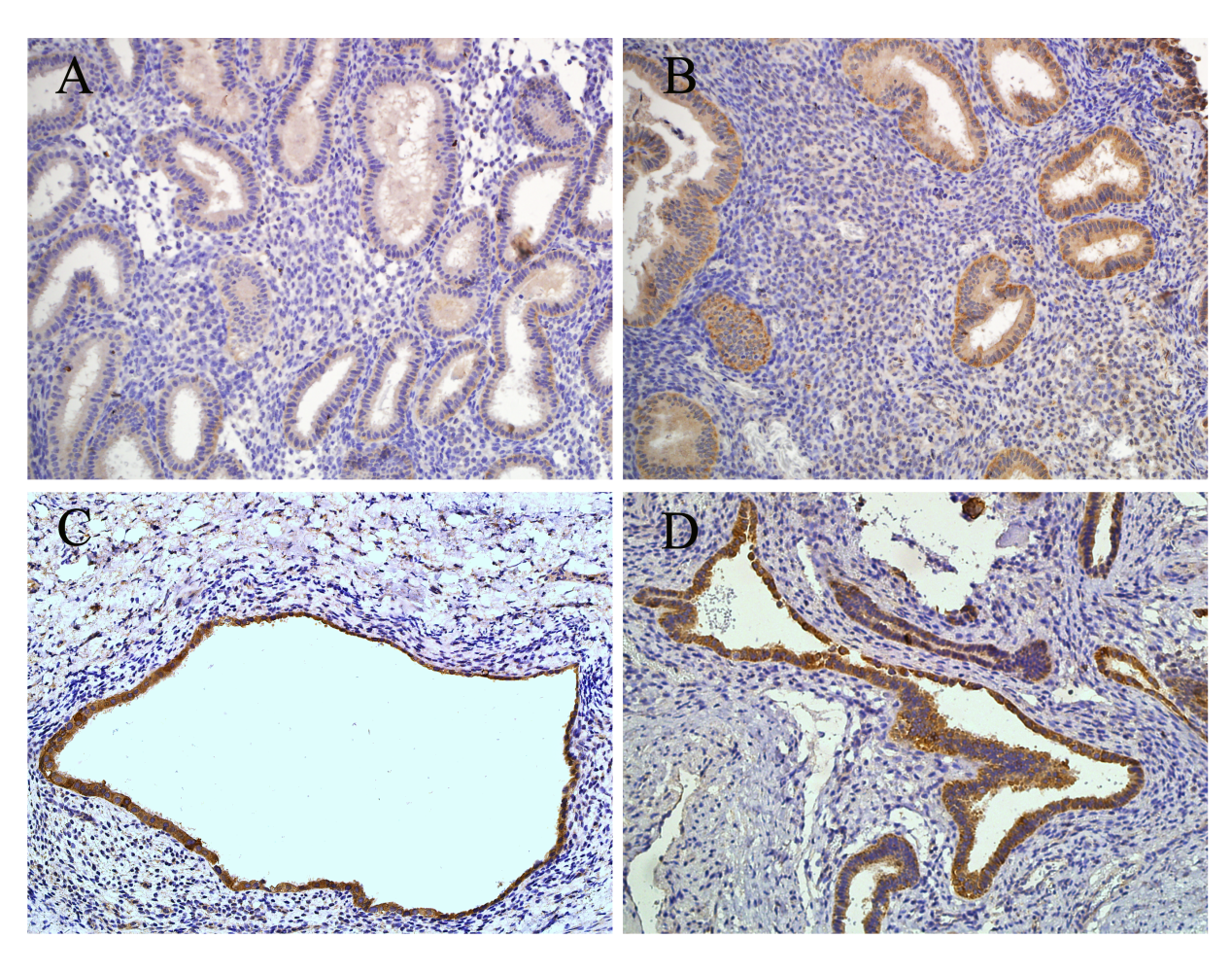

Supplement: S12 Fig — PEM: peritoneal endometriosis; USL-EM: deep infiltrating endometriosis of uterosacral ligament. (immunohistochemical stain, 200 × magnification) (DOCX) [file pone.0146027.s018.docx]

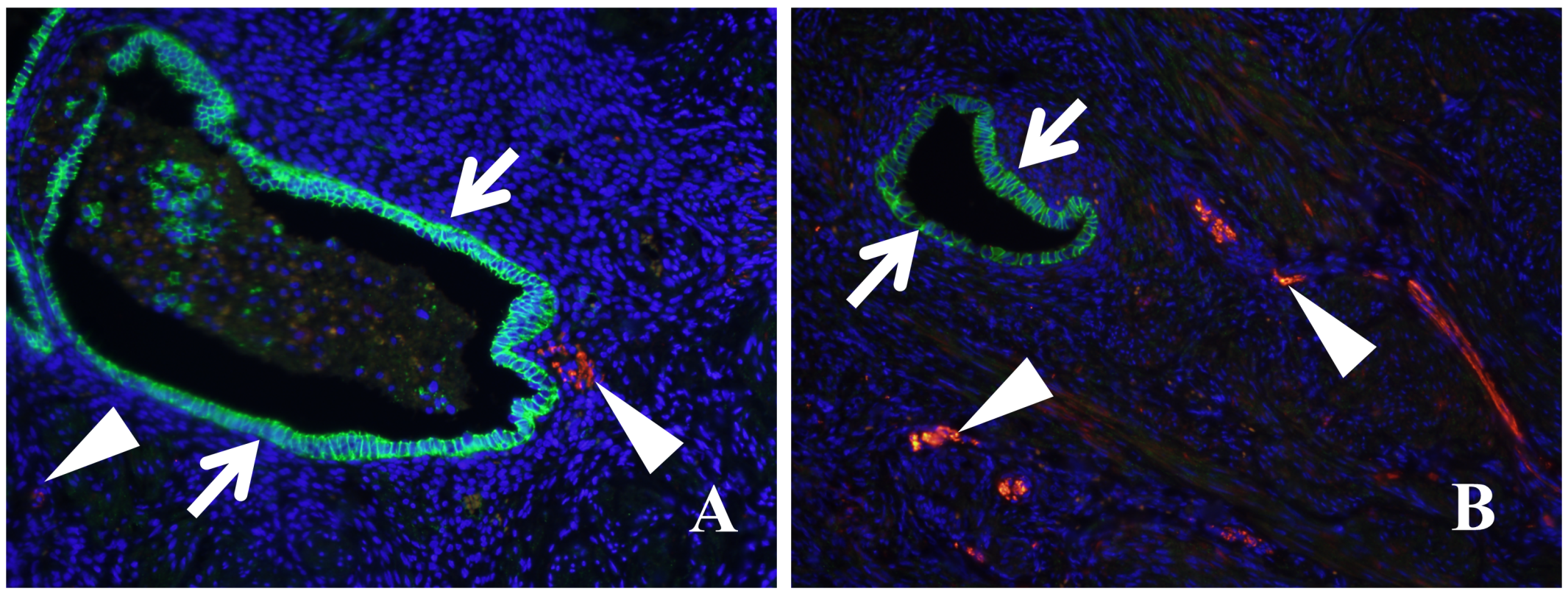

Supplement: S13 Fig — A: Merged image of double staining of both Sema 3A and TH (white arrow: peritoneal endometriotic glands, Sema 3A positive stained in green; white triangle: endometriosis-associated sympathetic nerve, TH positive stained in orange yellow; nuclei were stained in blue by DAPI staining). B: Merged image of double staining of both Sema 3A and PGP 9.5 (white arrow: endometriotic glands of deep infiltrating endometriosis of uterosacral ligament, Sema 3A positive stained in green; white triangle: PGP 9.5 positive stained endometriosis-associated nerve, orange yellow; nuclei were stained in blue by DAPI staining). Original magnification: 200×. TH: Tyrosine hydroxylase. (DOCX) [file pone.0146027.s019.docx]
